# Systemic Inflammatory Response to Smoking in Chronic Obstructive Pulmonary Disease: Evidence of a Gender Effect

**DOI:** 10.1371/journal.pone.0097491

**Published:** 2014-05-15

**Authors:** Rosa Faner, Nuria Gonzalez, Tamara Cruz, Susana Graciela Kalko, Alvar Agustí

**Affiliations:** 1 Fundació Privada Clínic per a la Recerca Biomèdica, Barcelona, Spain; 2 Institut d'investigacions Biomèdiques August Pi i Sunyer (IDIBAPS), Barcelona, Spain; 3 CIBER Enfermedades Respiratorias (CIBERES), Barcelona, Spain; 4 Bioinformatics Core Facility, IDIBAPS, Barcelona, Spain; 5 Thorax Institute, Hospital Clinic, University of Barcelona, Barcelona, Spain; 6 Fundació de Investigació Sanitaria Illes Balears (FISIB), Mallorca, Spain; University of California San Francisco, United States of America

## Abstract

**Background:**

Tobacco smoking is the main risk factor of chronic obstructive pulmonary disease (COPD) but not all smokers develop the disease. An abnormal pulmonary and systemic inflammatory response to smoking is thought to play a major pathogenic role in COPD, but this has never been tested directly.

**Methods:**

We studied the systemic biomarker and leukocyte transcriptomic response (Affymetrix microarrays) to smoking exposure in 10 smokers with COPD and 10 smokers with normal spirometry. We also studied 10 healthy never smokers (not exposed to smoking) as controls. Because some aspects of COPD may differ in males and females, and the inflammatory response to other stressors (infection) might be different in man and women, we stratified participant recruitment by sex. Differentially expressed genes were validated by q-PCR. Ontology enrichment was evaluated and interaction networks inferred.

**Results:**

Principal component analysis identified sex differences in the leukocyte transcriptomic response to acute smoking. In both genders, we identified genes that were differentially expressed in response to smoking exclusively in COPD patients (COPD related signature) or smokers with normal spirometry (Smoking related signature), their ontologies and interaction networks.

**Conclusions:**

The use of an experimental intervention (smoking exposure) to investigate the transcriptomic response of peripheral leukocytes in COPD is a step beyond the standard case-control transcriptomic profiling carried out so far, and has facilitated the identification of novel COPD and Smoking expression related signatures which differ in males and females.

## Introduction

Tobacco smoking is the major risk factor for Chronic Obstructive Pulmonary Disease (COPD) [1]. Yet, only a proportion of smokers, so called “susceptible smokers”, develop the disease [2]. The genetic and epigenetic background of each smoker is likely to regulate the type and intensity of his/her inflammatory response to smoking [1], [3]–[5]. In “susceptible smokers”, this response is thought to be “enhanced”, both in the lungs [6] and in the systemic circulation [7], and is believed to drive disease progression [1], [6]. However, despite the wide acceptance of this notion [1], no previous study has actually studied the “response” to smoking (i.e., the specific inflammatory changes that occur before and after smoking) in susceptible smokers (i.e., patients with COPD) and resistant smokers (i.e., smokers with normal spirometry). Rather, available evidence compares a number of inflammatory markers in these two groups of smokers “after” many years of smoking exposure [6].

To address this gap in knowledge, we compared a number of systemic inflammatory biomarkers and the transcriptome of circulating leukocytes, before and after smoking in susceptible (COPD patients) and resistant smokers. We hypothesized that smoking exposure will induce a different inflammatory signature, at the cellular, protein and/or transcriptome levels, in these two groups of smokers. Importantly, because several previous reports suggest that there may be significant gender differences in the natural history of COPD [8]–[10] and some experimental observations show that the leukocyte transcriptional response to other acute stressors (infection) is different in males and females [11], we recruited participants stratified by sex.

## Methods

Data S1 presents an extended explanation of the Methods used.

### Design, Participants and Ethics

In this prospective and controlled study, we included 30 volunteers stratified by smoking history, presence of COPD [12] and sex. All COPD patients were clinically stable. The Ethics Committee the Hospital Clinic de Barcelona approved the study protocol and all participants signed their informed consent.

### Acute Smoking Exposure Test (ASET)

Current smokers were asked to refrain from smoking for, at least, 8 hours before the ASET. Exhaled carbon monoxide concentration was measured (SC01-STK, CO SmokeCheck Monitor, Care Fusion, San Diego, US) to confirm smoking abstinence. All participants fasted overnight. A catheter was inserted in a peripheral vein and, based on previous studies in healthy subjects [13], [14], smokers were asked to smoke 2 cigarettes (Nobel tar content 9 mg, nicotine 0.7 mg and carbon monoxide 9 mg, Altadis S.A. Spain) in 30 minutes under the direct supervision of an investigator. Venous blood samples were obtained before (T0), 30 (T30) and 180 minutes (T180) after smoking. Plasma and buffy coat were separated immediately by centrifugation at 400 g×6 minutes. Plasma was stored at −70°C until analysis. Erythrocytes were lysed, leukocytes washed, lysed in RLT buffer and stored at −70°C for later RNA extraction. Participants remained at rest for the duration of the test.

### Measurements

#### Forced spirometry

Forced spirometry was measured according to international recommendations [15]. Reference values correspond to a Mediterranean population [16].

#### Circulating inflammatory markers

Total white blood cells counts were quantified with an ADVIA-2120 system (Siemens, Germany). The plasma concentration of C-Reactive Protein (CRP) was determined by ultra-sensitive quantitative turbidimetric test (Bayer Diagnostics, Germany), and those of interleukin (IL)-6 (IL-6) and IL-8 by high sensitivity ELISA (Invitrogen, USA). Blood carboxyhemoglobin levels were determined with a Rapid Point 500 Blood-Gas-System (Siemens, Germany).

#### Leukocyte mRNA profile

RNA was extracted using RNeasy Mini Kit (Qiagen, Valencia, US) following manufacturer instructions. RNA integrity was assessed with an Agilent 2100 Bionalyzer (Agilent Technologies). All samples had a RIN above 8. The 60 RNA samples were hybridized to Affymetrix HG-U219 array plate, which enables the performance of up to 96 arrays at one time, following Affymetrix's protocols. Microarray data has been deposited in the GEO NCBI database (accession code GSE55962).

#### Array validation by real time PCR

Selected genes were subjected to real-time quantitative PCR (q-PCR) for independent confirmation of relative expression levels in each group using Taqman assays on demand, actin as a housekeeping gene, and relative quantification was performed with the 2^(ΔCP)^ method [17].

### Data analysis

qPCR and systemic biomarker results are summarized as mean ± standard deviation, proportions or range, as appropriate. Clinical variables were compared across groups using unpaired tests (Mann-Whitney, Kruskal-Wallis). Paired tests (Wilcoxon) were used to compare T0 and T180 results within each group.

Differential gene expression was investigated using Rank Prod [18]; probe-sets with FDR (False Discovery Rate) values<0.05 were considered significant. Principal components analysis (PartekH Pro) was used to explore visually global effects on transcription. DAVID (www.david.abcc.ncifcrf.gov) [19], [20] was used to estimate the functional over-representation of the significant genes in the Gene Ontology Biological Process database (www.geneontology.org). Interaction networks were constructed using Ingenuity Pathways Analysis (IPA, www.ingenuity.com) based on the Ingenuity Pathways Knowledge Base (IPKB) [21]. Cytoscape (www.cytoscape.org) was used to analyze and display correlations results graphically [22], [23].

## Results

### Participant Characteristics

Participants showed the differences in sex, smoking history and lung function expected from the stratified recruitment strategy of the study (Table 1). Of note: (*1*) age was similar in COPD patients and smokers with normal spirometry; (*2*) airflow limitation in COPD patients ranged from moderate to severe. Despite that it was similar in males and females with COPD, the latter were older (p = 0.02) and tended to have smoked less (p = 0.07); and (*3*) cumulative smoking exposure was higher in males with COPD than in male controls (p = 0.01), although this was not observed in females.

**Table 1:** Characterization of participants by sex (mean±SD).

	COPD	Smokers	Non Smokers
N (male/female)	9 (5/4)	11 (6/5)	10 (5/5)
Age , years			
Male	55±5	51±2	55±6
Female	68±3*	58±8	58±5
BMI, Kg/m^2^			
Male	26.4±3.4	25.0±1.7	26.4±1.1
Female	29.2±1.6	24.3±4.2	27.5±6.2
Pack/year			
Male	70±21§	24±14	0
Female	40±11	34±8	0
FEV1/FVC, %			
Male	61±4.9§	77±4	81±2.2
Female	53±4.9§	74±2.7	78±3.9
FEV1, % reference			
Male	64±12§	93±12	96±12
Female	63±14	84±11	88±12

Asterisk indicate p<0.05 between COPD males and females, § indicates p<0.05 between COPD patients and smoker controls.

### Biomarker Response to Smoking

**Figure 1:**
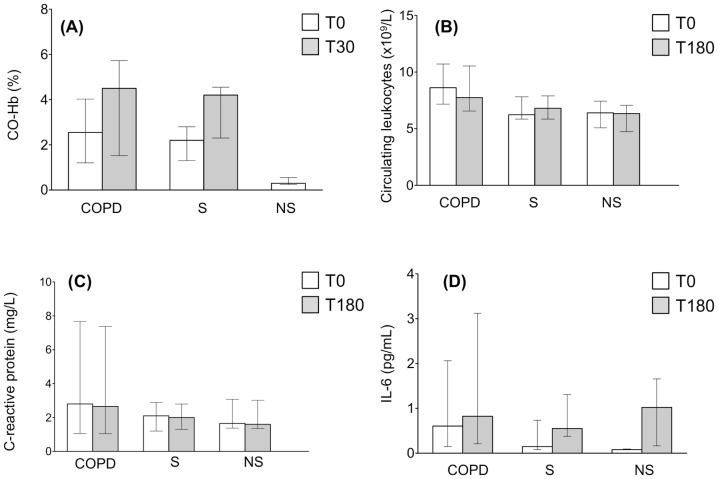
Median (and IQR) values of blood carboxy-hemoglobin (*panel A*), circulating leukocytes (*panel B*), C-reactive protein serum levels (*panel C*) and IL-6 (*panel D*) in COPD patients, smoker (S) and non-smoker (NS) controls, before (T0) and 30 min. (T30) or 180 min. (T180) later. For further explanations, see text.

**Figure 2:**
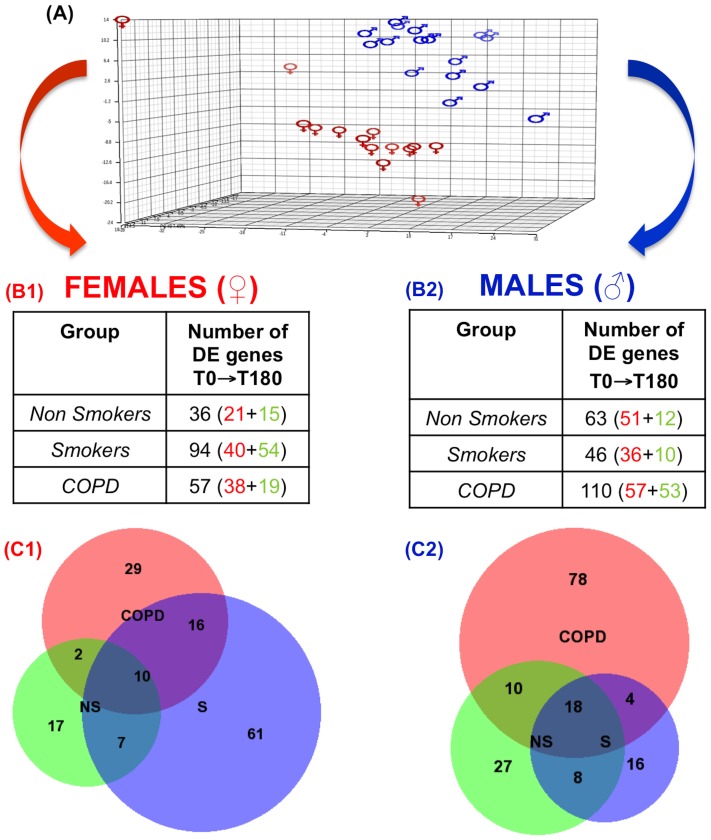
*Panel A*: Principal component analysis (PCA) of those genes with the largest variability of expression values across the experimental groups (COPD patients, smokers (S) and non-smokers (NS)) at baseline (T0). Because the sex effect observed, further analysis was stratified by sex (right and left columns.*) Panels B1 (females) and B2 (males)* present the number of differentially expressed (DE) genes from T0 to T180 in the three groups of participants studied. Red and green figures indicate up and down-regulation, respectively. *Panels C1 (females) and C2 (males)* show a BioVenn diagram (www.cmbi.ru.nl/cdd/biovenn) with the number of DE genes shared between COPD, S and NS controls. For further explanations, see text.

At baseline, carboxyhemoglobin levels were lower (p<0.001) in non-smokers (NS) than in smokers with normal spirometry (S) or with COPD, and they increased further thirty minutes after smoking (Figure 1, panel A). Circulating leukocyte counts at T0 were higher (p = 0.02) in COPD than in controls, and they did not change at T180 in any group (Figure 1, panel B); likewise, we did not observed any significant change after smoking in circulating neutrophils, lymphocytes (CD3+), CD4+ T cells, CD8+ T, CD19+ B cells or NK populations (data not shown). Mean CRP (Figure 2, panel C) and IL-6 levels (Figure 1, panel D) were within the normal range [7] in all groups and time points. IL-8 values were below the lower detection limit in all participants, both before and after smoking (data not shown). Finally, we did not observe significant sex differences in any of these biomarkers, either before or after smoking.

### Leukocyte Transcriptomic Response to Smoking

Principal component analysis (PCA) allows the visualization of differences within a multidimensional data set. Here, a PCA of the expression values of those genes that show the largest variability across all experimental groups (COPD, S and NS) at baseline demonstrated a clear segregation of the leukocyte transcriptome profile by sex (Figure 2, Panel A). Hence, further statistical differential analyses were performed separately by gender, as previously described [24]. Figure 2 (Panels B1 and B2) presents the number of differentially expressed (DE) genes between T0 and T180 (red and green figures indicate up or down DE, respectively) in NS, S and COPD stratified by sex (Table S1 presents the complete list of DE genes). Overall, transcriptomic changes were of moderate intensity (log ratio ranged between 0.95 and 0.4). Unsupervised clustering of DE genes in COPD segregated T0 and T180 samples adequately, both in females (n = 57) and males (n = 110) (Figure S1, panels A and B, respectively) indicating that the Rank Prod statistical analysis used here identified clear gene signatures associated with acute smoking exposure.

#### Identification of COPD and Smoking related genes

BioVenn diagrams in Figure 2 present the number of DE genes from T0 to T180 that were shared (or not) between groups (NS, S and COPD), both in females (Panel C1) and males (Panel C2). Given that NS did not smoke, genes that were DE from T0 to T180 in this group (36 in females and 63 in males) were consider to represent time-dependent changes, and were therefore excluded from any further analysis investigating the response to smoking in S or COPD. Likewise, we operationally considered that those genes that were DE in response to smoking only in COPD patients represent *COPD related* genes, whereas those genes DE in response to smoking only in smokers with normal spirometry represent *Smoking related genes*. Accordingly, we identified 29 COPD related genes in females (78 in males), and 61 Smoking genes in females (16 in males) (Figure 2, Panels C1 and C2, respectively). Table 2 lists the top 10 susceptibility and resistance genes ordered by log ratio after smoking, direction of change and sex.

**Table 2:** Top 10 COPD a) and Smoking b) related genes ordered by log ratio after smoking, direction of change (up vs. down-regulation) and sex.

a)	COPD males				COPD females			
**Up-regulated genes**	**n#**	**symbol**	**Gene Description**	**Log ratio**	**n#**	**symbol**	**Gene Description**	**Log ratio**
	1	SPAG9	Sperm associated antigen 9	0.69	1	ELMOD2	ELMO/CED-12 domain containing 2	0.71
	2	ZNF441	Zinc finger protein 441	0.64	2	ZFAND6	Zinc finger, AN1-type domain 6	0.71
	3	BIRC3	Baculoviral IAP repeat containing 3	0.63	3	SNX13	Sorting nexin 13	0.7
	4	S1PR1	Sphingosine-1-phosphate receptor 1	0.62	4	PAFAH1B1	Platelet-activating factor acetylhydrolase 1b	0.66
	5	MGAT4A	Mannosyl (alpha-1,3-)-glycoprotein beta-1,4-N-acetylglucosaminyltransferase, isozyme A	0.61	5	TMEM170B	Transmembrane protein 170B	0.66
	6	CLK1	CDC-like kinase 1	0.6	6	RABEP1	Rabaptin, RAB GTPase binding effector protein 1	0.62
								
	7	KLHL9	Kelch-like family member 9	0.6	7	CCPG1	Cell cycle progression 1	0.61
	8	SLFN5	Schlafen family member 5	0.59	8	TMEM41B	Transmembrane protein 41B	0.6
	9	ARRDC3	Arrestin domain containing 3	0.59	9	CLK1	CDC-like kinase 1	0.58
	10	SOCS4	Suppressor of cytokine signaling 4	0.59	10	MALAT1	Metastasis associated lung adenocarcinoma transcript 1	0.53
**Down-regulated genes**	**n#**	**symbol**	**Gene Description**	**Log ratio**	**n#**	**symbol**	**Gene Description**	**Log ratio**
	1	HLX	H2.0-like homeobox	−0.79	1	CXCL1	Chemokine (C-X-C motif) ligand 1	−0.94
	2	TRIB1	Tribbles homolog 1	−0.79	2	FOS	FBJ osteosarcoma oncogene	−0.68
	3	CHST11	Carbohydrate (chondroitin 4) sulfotransferase 11	−0.72	3	NT5C2	5′-nucleotidase, cytosolic II	−0.59
	4	FLJ36031	CCDC71L coiled-coil domain containing 71-like	−0.59	4	BCL2A1	BCL2-related protein A1	−0.56
	5	STK17B	Serine/threonine kinase 17 b (apoptosis-inducing)	−0.58	5	RBBP6	Retinoblastoma binding protein 6	−0.54
	6	CD24	CD24 molecule	−0.58	6	WIPF1	WAS/WASL interacting protein family, member 1	−0.53
								
	7	SGK1	Serum/glucocorticoid regulated kinase 1	−0.57	7	JUN	Jun proto-oncogene	−0.52
	8	IL1R2	Interleukin 1 receptor, type II	−0.56	8	DNAJB1	DnaJ (Hsp40) homolog, subfamily B, member 1	−0.52
								
	9	BCL2A1	BCL2-related protein A1	−0.52	9	RBM6	RNA binding motif protein 6	−0.51
	10	KYNU	Kynureninase	−0.52	10	TRA2A	Transformer 2 alpha homolog (Drosophila)	−0.51

b)	Smoker males				Smoker females			
		
**Up-regulated genes**	**n#**	**symbol**	**Gene Description**	**Log ratio**	**n#**	**symbol**	**Gene Description**	**Log ratio**
	1	IL18R1	Interleukin 18 receptor 1	0.48	1	REPS2	RALBP1 associated Eps domain containing 2	0.71
	2	FGFBP2	Fibroblast growth factor binding protein 2	0.48	2	FOSL2	FOS-like antigen 2	0.63
	3	XYLT1	Xylosyltransferase I	0.45	3	CDA	Cytidine deaminase	0.61
	4	ZNF564	Zinc finger protein 564	0.43	4	CPEB2	Cytoplasmic polyadenylation element binding protein 2	0.6
	5	ADH5	Alcohol dehydrogenase 5	0.38	5	PKN2	Protein kinase N2	0.59
	6	SIPA1L2	Signal-induced proliferation-associated 1 like 2	0.36	6	ZNF267	Zinc finger protein 267	0.58
					7	TNFSF14	Tumor necrosis factor (ligand) superfamily, member 14	0.57
					8	DHRS9	Dehydrogenase/reductase (SDR family) member 9	0.57
					9	TBX21	T-box 21	0.57
					10	ABCG1	ATP-binding cassette, sub-family G (WHITE), member 1	0.55
**Down-regulated genes**	**n#**	**symbol**	**Gene Description**	**Log ratio**	**n#**	**symbol**	**Gene Description**	**Log ratio**
	1	HSPH1	Heat shock 105 kDa/110 kDa protein 1	−0.69	1	DDIT4	DNA-damage-inducible transcript 4	−0.77
	2	CPSF6	Cleavage and polyadenylation specific factor 6, 68 kDa	−0.52	2	KLF9	Kruppel-like factor 9	−0.72
	3	CLC	Charcot-Leyden crystal galectin	−0.5	3	ETS1	V-ets avian erythroblastosis virus E26 oncogene homolog 1	−0.61
	4	DDX17	DEAD (Asp-Glu-Ala-Asp) box helicase 17	−0.46	4	ANKRD10	Ankyrin repeat domain 10	−0.59
	5	DDX5	DEAD (Asp-Glu-Ala-Asp) box helicase 5	−0.43	5	PPIP5K2	Diphosphoinositol pentakisphosphate kinase 2	−0.53
	6	SRSF10	Serine/arginine-rich splicing factor 10	−0.41	6	AHR	Aryl hydrocarbon receptor	−0.52
	7	POU2AF1	POU class 2 associating factor 1	−0.39	7	KLF10	Kruppel-like factor 10	−0.52
	8	SP100	SP100 nuclear antigen	−0.37	8	TLE4	Transducin-like enhancer of split 4, homolog of Drosophila E(spl)	−0.51
	9	CEP350	Centrosomal protein 350 kDa	−0.36	9	PABPN1	Poly(A) binding protein, nuclear 1	−0.49
	10	BAX	Bcl-2-associated X protein	−0.28	10	ARGLU1	Arginine and glutamate rich 1	−0.47

Additionally, we investigated if baseline differences in gene expression between groups (S vs. COPD) and/or sex (male vs. female) influence the transcriptional response to smoking. Results are explained in detail in the data S1 (and Tables S2 and S3) but, by and large, DE at baseline was only marginally related to the transcriptomic response to smoking.

#### COPD and Smoking related genes shared by males and females

We identified five COPD related genes shared by males and females: (*1*) WIPF1 (mean log ratio = −0.479), an important regulator of the immunological synapsis formation and T cell activation[25]; (*2*) BCL2A1 (mean log ratio = −0.538), that regulates p53tumor suppression induced apoptosis [26]; (*3*) SGK1 (mean log ratio = −0.512), whose up regulation is involved in hypertension, obesity, diabetes, thrombosis, stroke, fibrosing disease, infertility and tumor growth, and its inhibition mediates skeletal muscle homeostasis and function [27]; (*4*) ZNF397 (mean log ratio = 0.551), thought to have a role in centromere formation and gene transcription [28]; and, finally, (*5*) CLK1 (mean log ratio = 0.589), an alternative splicing factor induced by hypoxia [29].

Similarly, we identified two Smoking related genes shared by males and females: POU2AF1 (log ratio = −0.40), a nuclear factor responsible of immunoglobulin transcription regulation [30], and XYLT1 (log ratio = 0.45), the initial enzyme responsible of GAG biosynthesis that has been associated with tissue remodeling [31].

#### Gene Ontologies Associated with COPD and Smoking related genes

We used DAVID [19], [20] to identify enriched gene ontologies (% FDR<25) associated with the COPD susceptibility and resistance genes identified above. In females, ontologies enriched in *COPD related genes* (n = 29) included genes that were mostly down regulated (Table 3). Of note, MLL5, JUN and FOS, were involved in most of the ontologies. It is known that FOS and JUN dimerize to form the transcription factor complex AP-1 a key player in the regulation of several biological processes [32]. In keeping with this we observed that enriched ontologies included regulation of transcription control, metabolism of nucleic acids and biosynthetic processes (Table 3) as well as ontologies related with the immune system, such as SMAD signaling and response to extracellular stimulus. On the other hand, ontologies enriched in *Smoking related genes* in females (n = 61) included both up and down-regulated genes, and were related with the immune system, cell cycle, cellular growth, apoptosis and cell locomotion (Table 4). The former included genes involved in T cell activation (ETS1 and TNFSF14) and antigen presentation control (CYBB and CST7).

**Table 3:** Gene ontology enrichment analysis, by sex. Females ontologies/genes associated with COPD.

Term	Genes	% FDR	p value
GO:0009991 response to extracellular stimulus	FOS, MLL5, JUN, KLF4	7	5.20E-03
GO:0045893 positive regulation of transcription, DNA-dependent	FOS, MLL5, JUN, RBM4, KLF4	9	6.74E-03
GO:0051254 positive regulation of RNA metabolic process	FOS, MLL5, JUN, RBM4, KLF4	9	6.94E-03
GO:0060395 SMAD protein signal transduction	FOS, JUN	12	0.01
GO:0045941 positive regulation of transcription	FOS, MLL5, JUN, RBM4, KLF4	15	0.01
GO:0010628 positive regulation of gene expression	FOS, MLL5, JUN, RBM4, KLF4	17	0.01
GO:0007611 learning or memory	FOS, JUN, ***PAFAH1B1***	18	0.01
GO:0045935 positive regulation of nucleobase, nucleoside, nucleotide and nucleic acid metabolic process	FOS, MLL5, JUN, RBM4, KLF4	21	0.02
GO:0051173 positive regulation of nitrogen compound metabolic process	FOS, MLL5, JUN, RBM4, KLF4	23	0.02
GO:0010557 positive regulation of macromolecule biosynthetic process	FOS, MLL5, JUN, RBM4, KLF4	24	0.02

Bold italic text indicates up regulated genes and normal text indicates down regulated genes in response to smoking.

**Table 4:** Gene ontology enrichment analysis, by sex. Females ontologies/genes associated with Smoking.

Term	Genes	% FDR	p value
GO:0006955 immune response	CYBB, POU2AF1, ***IL18RAP, CST7*** *,* ETS1, BCL2, LILRB4, ***TGFBR3, TNFSF14, CLEC4D***	0.7	4.50E-04
GO:0043065 positive regulation of apoptosis	***ADRB2*** *,* ETS1, ***MMP9*** *,* KLF10, BCL2, ***TNFSF14, FADD, RRM2B***	0.9	5.70E-04
GO:0043068 positive regulation of programed cell death	***ADRB2*** *,* ETS1, ***MMP9*** *,* KLF10, BCL2, ***TNFSF14, FADD, RRM2B***	0.9	6.00E-04
GO:0010942 positive regulation of cell death	***ADRB2*** *,* ETS1, ***MMP9*** *,* KLF10, BCL2, ***TNFSF14, FADD, RRM2B***	0.9	6.10E-04
GO:0051270 regulation of cell motion	ETS1, IL6ST, ***MMP9*** *,* BCL2, ***TGFBR3***	6	4.00E-03
GO:0051272 positive regulation of cell motion	ETS1, IL6ST, ***MMP9*** *,* BCL2	7	4.00E-03
GO:0042127 regulation of cell proliferation	***ADRB2, FOSL2*** *,* ETS1, IL-6ST, KLF10, BCL2, ***CHST11, TGFBR3, ING1***	7	5.00E-03
GO:0042981 regulation of apoptosis	***ADRB2*** *,* ETS1, ***MMP9*** *,* KLF10, BCL2, *CHST11, * ***TNFSF14, FADD, RRM2B***	8	5.00E-03
GO:0043067 regulation of programmed cell death	***ADRB2*** *,* ETS1, ***MMP9*** *,* KLF10, BCL2, *CHST11, * ***TNFSF14, FADD, RRM2B***	8	6.00E-03
GO:0010941 regulation of cell death	*ADRB2,* ETS1, ***MMP9*** *,* KLF10, BCL2, *CHST11, * ***TNFSF14, FADD, RRM2B***	9	6.00E-03
GO:0045926 negative regulation of growth	***ADRB2*** *,* BCL2, **CDA, ING1**	9	6.00E-03
GO:0005976 polysaccharide metabolic process	***XYLT1*** *,* IL6ST, ***CHST11, MGAM***	10	6.00E-03
GO:0048643 positive regulation of skeletal muscle tissue development	***ADRB2*** *,* BCL2	15	0.01
GO:0045844 positive regulation of striated muscle development	***ADRB2*** *,* BCL2	19	0.01
GO:0048636 positive regulation of muscle development	***ADRB2*** *,* BCL2	19	0.01
GO:0051094 positive regulation of developmental process	***ADRB2*** *,* ETS1, IL-6ST, KLF10, BCL2	21	0.01

Bold italic text indicates up regulated genes and normal text indicates down regulated genes in response to smoking.

On the other hand, in males *COPD related genes* (n = 78) were related to immune system, cellular locomotion and cellular activation ontologies (Table 5). Interestingly, some of these ontologies were similar to those identified in females as *Smoking related* ontologies but with an opposite DE sign (up vs. down-regulation). For instance, as shown in Table 5, genes involved in cell migration were mainly activated in response to smoking in males with COPD but mostly repressed in female S with normal lung function. The ontology enrichment analysis of *Smoking related genes* in males failed to obtain significant terms because of the small number of genes identified (n = 16) (Figure 2, panel C2).

**Table 5:** Gene ontology enrichment analysis, by sex. Males ontologies/genes associated with COPD.

Term	Genes	% FDR	p value
GO:0006955 immune response	***GPR183*** *,* IL1R2, ***SH2D1A*** *,* KYNU, CLEC4E, ***THEMIS*** *,* SNCA, ***EOMES, TGFBR3*** *,* CLEC4D, CD24	1	9.00E-04
GO:0040012 regulation of locomotion	***SPAG9, S1PR1*** *,* SNCA, ***TGFBR3, CBLL1*** *,* TRIB1	3	1.71E-03
GO:0030334 regulation of cell migration	***SPAG9, S1PR1, TGTBR3, CBLL1*** *,* TRIB1	10	7.06E-03
GO:0051270 regulation of cell motion	***SPAG9, S1PR1, TGTBR3, CBLL1*** *,* TRIB1	16	1.00E-02
GO:0030098 lymphocyte differentiation	***GRP183, THEMIS, EOMES*** *,* CD24	16	1.00E-02
GO:0001824 blastocyst development	***HOPX, EOMES, TGFBR3***	23	2.00E-02

Bold italic text indicates up regulated genes and normal text indicates down regulated genes in response to smoking. Analysis could not be done for ontologies/genes associated with COPD resistance in males due to the small number of DE genes (n = 16) identified in smokers with normal spirometry.

#### Expression Correlation Networks of COPD and Smoking related Genes

**Figure 3:**
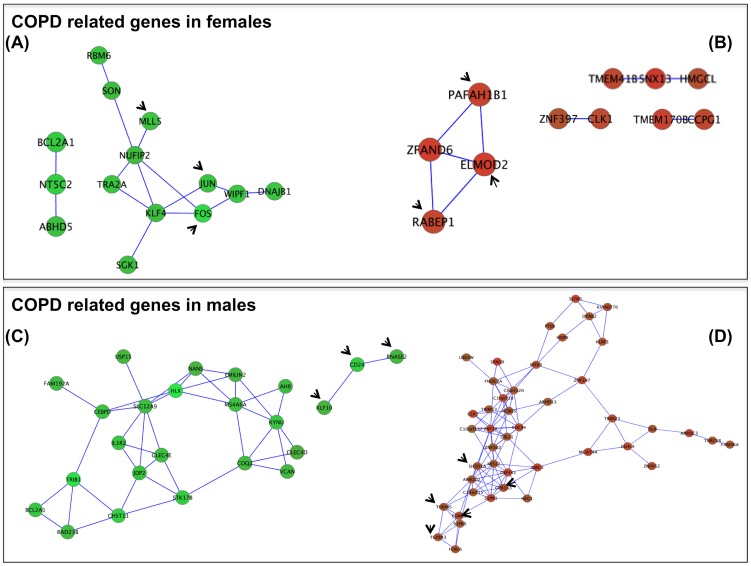
Expression correlation networks (Pearson r≥0.8) of COPD related genes in females (*Panels* A and B) and males (*Panels* C and D). Red and green figures indicate up and down-regulation of expression, respectively. Arrows point genes forming functional clusters identified in the network. For further explanations, see text.

**Figure 4:**
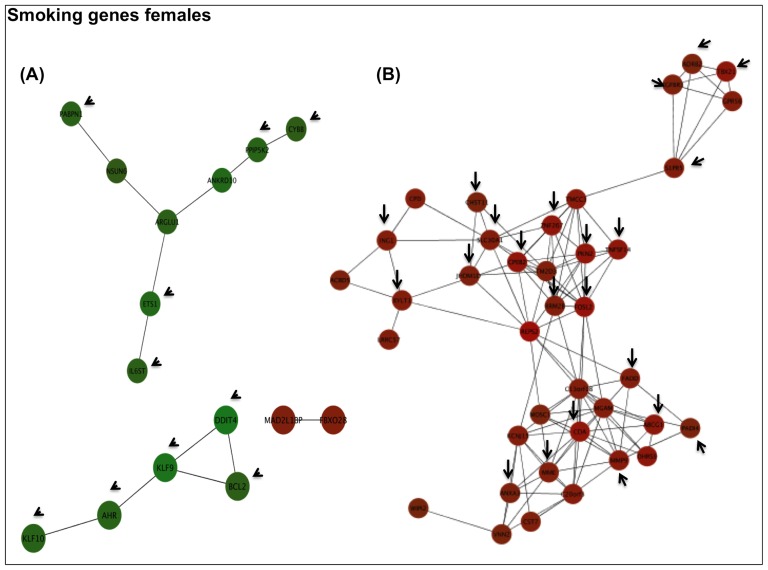
Expression correlation networks (Pearson r≥0.8) of Smoking genes in females. Arrows indicate genes identified by gene ontology analysis in the network. Red and green indicate up and down-regulation, respectively. For further explanations, see text.

We used Cytoscape (plug-ins Expression Correlation and Bingo) [22] to explore if the expression of the COPD and *Smoking related* genes identified above were correlated (Pearson r≥0.8) and form networks that have coordinated functionality. We found that, in females, *COPD related genes* indeed formed several correlation networks of different size (Figure 3, panels A and B). Some of them included genes that form functional cores (arrows) because they belong to the same ontologies. For instance, MLL5, JUN and FOS define a functional core of (repressed) genes (Panel A) that belong to three ontologies: GO60395 (SMAD protein signal transduction, p value = 0.0001), GO6306 (DNA methylation, p value  = 0.0003) and GO6305 (DNA alkylation, p value = 0.0003). On the other hand, PAFAH1B1, ELMOD2 and RABEP1 formed a functional core of up-regulated susceptibility genes (panel B) participating in GO16044 (cellular membrane organization, p = 0.001). Overall, these results confirm, expand and support our previous analysis using DAVID. Figure 4 depicts the correlation networks of *Smoking related genes* in females. Two down-regulated networks were identified containing ontologies related to GO44237 cellular metabolic process (p = 0.015) and GO42221 response to chemical stimulus (p = 0.0005) among others. A large positive correlation network was identified, containing several ontologies as GO7275 multicellular organismal development (p = 0.0039) and GO50794 regulation of cellular process (p = 0.006).

In males, *COPD related genes* form (Figure 3): (*1*) a functional core of repressed genes (arrows, Panel C) that belong to “Response to chemical stimulus” (GO42221, p = 0.001); and, (*2*) a cluster (arrows) of up-regulated genes (Panel D) involved in GO2376 (Immune system process, p = 0.007) and GO30097 (Hemopoiesis, p = 0.0002). The network analysis of *Smoking* in males was not possible because of the low number of genes identified (n = 16).

#### Interaction Network Analysis of COPD and Smoking related genes

**Figure 5:**
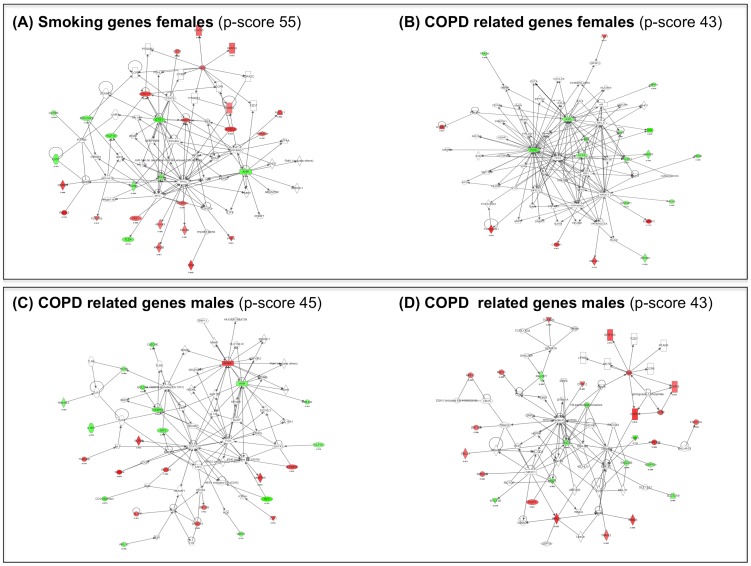
Putative interaction networks identified by Ingenuity Pathway Analysis (IPA)[21], and their corresponding scores, in female smoker controls COPD related in females (***Panel A)***, Smoking in females (***Panel B)*** and COPD related in males (***Panels C*** and ***D***). For further explanations, see text.

To infer interaction networks of *COPD* and *Smoking related* genes we used Ingenuity Pathways Analysis (IPA), a database of known molecule interactions [21]. In females, from the 61 *Smoking* genes (Figure 2, panel C1) IPA identified one significant network (IPA p-score 55) (Figure 5, panel A) whose main functional terms included organismal injury and abnormalities, cell death and survival and cell morphology. We selected the ETS gene, a central hub in this network, for independent validation by real time PCR (qPCR); the later confirmed the observed microarrays transcriptional change (Figure S2). Likewise, from the 29 *COPD related* genes identified in females (Figure 2, panel C1), IPA identified a network (p-score 43) enriched in cell death and survival, lipid metabolism and small molecule biochemistry functions (Figure 5, panel B). In this network, we selected FOS and CXCL1 for independent qPCR validation; results (Figure S2) confirmed changes in both CXCL1 (p = 0.01) and FOS (p = 0.07).

The low number of *Smoking* genes (n = 16) identified in males did not allow obtaining significant networks in the IPA analysis. By contrast, from the 78 *COPD related* genes identified in males (Figure 2, panel C2), IPA identified two large networks. The first one (IPA p-score 45) included infectious disease, cellular function and maintenance, hematological system development and function as main functional terms (Figure 5, panel C). The second one (p-score 43) included DNA replication, recombination, cell cycle and cell morphology terms (Figure 5, panel D). From these two networks, we selected HLX, TRIB1 and BIRC3, known to be involved in cell activation, cell migration and apoptosis respectively, for qPCR validation (Figure S2).

## Discussion

Despite that an abnormal inflammatory response to smoking is believed to play a major pathogenic role in COPD [1], to our knowledge this is the first study to directly compare the systemic biomarker and transcriptomic leukocyte response to acute smoking exposure in susceptible (i.e. COPD patients) and resistant smokers (i.e., those with normal spirometry). By doing so, our results provide several novel and potentially relevant observations. First, we identified a differential transcriptomic pattern, both at baseline and in response to smoking, in males and females, whose implications go beyond COPD, as discussed below. Second, we identified a number of *COPD and Smoking related* genes (signatures), both in males and females, and we identified their enriched ontologies and gene interaction networks. Yet, due to the relatively small number of subjects studied, reproducibility in an independent and larger set of COPD patients and smokers is needed.

### Previous studies

Several previous studies have investigated the biological response to acute smoking exposure in healthy subjects [13], [14]. In fact, we designed the dose and timing of our ASET (see Methods) mostly based on these previous experiences. Yet, to our knowledge, no previous study has directly contrasted the transcriptomic response of circulating leukocytes to ASET in smokers with or without COPD. Bahr *et al* [33] compared the basal leukocyte transcriptomic pattern in COPD patients and non-smoker controls, without exposing subjects to smoking or considering potential sex differences. In our study, at baseline, we identified 19 DE genes also identified by Bahr *et al* [33] (C19orf59, ARHGAP18, C11orf75, CARD16, CLEC4D, FAIM3, FCRL3, GYG1, GZMM, HOPX, MAL, MAPK14, MCTP1, NELL2, RPS23, SAT1, SLC8A1, SNHG8, SPON2). Of note, MLL5, JUN, RABEP1 and ZFAND6 were identified in our study as *COPD related* genes in females and were also identified by Bhattacharya *et al* [34] as COPD associated genes in peripheral blood.

### Interpretation of findings

Leukocyte transcriptomics changes after acute smoking exposure were relatively low. This might not be surprising if this is framed within the natural history of COPD, which takes decades of smoking exposure to develop. In keeping with this, previous peripheral blood transcriptomic studies in COPD also reported small fold change differences [33], [34].

The fact that our study involved an intervention (ASET) in smokers with and without COPD, that it included never smokers (who served as a control for time-related biological effects), and that we stratified participants by sex prove to be key aspects of the experimental design and of paramount importance to identify different *COPD* and *Smoking related* signatures in males and female smokers. In males, the *COPD related signature* included ontologies related to the immune system (up-regulated) and cellular locomotion (up-regulated), whereas only a few number of genes (which limited ontology enrichment analysis) were associated with *Smoking*. By contrast, in females the *Smoking* signature included ontologies that were similar (but of opposite direction) to those identified as *COPD related* in males, and that their *COPD related* signature included down-regulation of the MLL5, FOS and JUN genes, all of them involved in the inflammatory response. Collectively, these observations suggest a tighter control of the inflammatory response to smoking in females.

How these sex differences in leukocyte transcriptomics might translate into the clinic is speculative but a number of clinically relevant gender differences have been long recognized in COPD [8]–[10]. For instance, previous studies of our group demonstrated that lung function decline with age was significantly higher in males than in females, both in healthy never smokers as well as in smokers with COPD [35]. A different regulation of the inflammatory response in males and females as that proposed here may well contribute to these observations. In fact, interestingly in the context of this discussion, systemic levels of inflammatory biomarkers appear reduced in females (vs. males) with COPD [36].

The relevance of these observations for other common smoking related diseases, such as cardiovascular disease [37]–[39] or lung cancer [40]–[42], deserves further investigation. In fact, recent publications support similar sex differences in other immune related diseases, such as multiple sclerosis [43], and it is well established that autoimmune diseases are particularly prevalent in females [44]. All in all, therefore, these observations call for a careful exploration and re-analysis of available COPD genetic data by gender.

Finally, we used several network analysis techniques to infer potential interactions among the identified *COPD and Smoking related* genes and thus to get further insight into the pathobiology of the disease. The expression correlation networks in conjunction with gene ontology identified functional cores of differentially expressed genes. The possible interaction networks between differentially expressed genes identify by IPA (on the basis of already know molecule interactions) showed good IPA p-scores and their main gene hub changes were validated by qPCR. In females, resistance genes networks included genes related to Gpcr signaling (SIPR5, GPR56), inflammation and Th1 responses (ETS-1, MMP9, TBX21), apoptosis (BCL2) and sensing of planar aromatic hydrocarbons that are present in tobacco (AHR), whereas the main hubs in their susceptibly gene networks include the repression of JUN and FOS, both involved also in the regulation of immune responses through the formation of the AP transcription factor. Overall, therefore, these observations suggest again a differential regulation of immune processes in females with or without COPD. On the other hand, in males, we identified two *COPD related* gene networks that involved, respectively, the response to planar aromatic hydrocarbons (hub gene AHR) and the immune response (hub gene RORA) on the one hand, and Gpcr signaling, ubiquitinization (TRIB1, RAD23B, USP-15) and apoptosis-autophagy (BIRC3, TRIM13, TRIM23) on the other, which were validated by qPCR. Interestingly, other studies have reported alterations in some of these processes in COPD [45]–[47].

### Strengths and Limitations

Our study has strengths and limitations. The fact that, despite the widespread assumption that COPD is characterized by an abnormal response to smoking, this is the first study to investigate this possibility specifically at the transcriptional level is a clear strength. Likewise, the use of an active experimental intervention (ASET) is a step beyond the standard transcriptomic case-control studies [48] and has in fact facilitated the identification of novel *COPD and Smoking related* genes in response to acute tobacco exposure. Finally, the use of network analysis has allowed the description of clear gender differences in the systemic inflammatory response to smoking in COPD patients and Smokers, an observation that may have implications beyond COPD in other smoking-related diseases. Among its limitations, we acknowledge that the transcriptomic changes after ASET were measured only in circulating leukocytes, so it is possible that changes in the pulmonary parenchyma may differ in type and/or intensity; needless to say that ethical and logistic difficulties involved in obtaining lung tissue samples before and after smoking are notorious. Finally, we included in the study 30 volunteers but, when stratified by presence of disease, smoking status and sex, each subgroup includes 5 individuals only. Due to the relatively small number of subjects studied, results need replication in an independent and larger cohort of COPD patients.

### Conclusions

This study shows that the transcriptomic response to smoke in circulating leukocytes is different in smokers with and without COPD, and also in males and females.

## Supporting Information

Figure S1 **Unsupervised clustering of DE genes in COPD segregated T0 and T180 samples adequately, both in females (n = 57) and males (n = 110) (panels A and B, respectively).**
(TIFF)

Figure S2 **qPCR validation of array results, RQ =  relative quantification of target gene mRNA to Actin mRNA calculated using the comparative CT method.** Central hub genes in IPA networks were selected for the validation; a) COPD males (HLX, TRIB1 and BIRC3), b) Healthy smoker females (ETS 1) and c) COPD females (CXCL1 and FOS1).
(TIFF)

Table S1 **Complete list of differentially expressed genes (DE) in COPD patients, smokers (S) and non-smokers (NS) stratified by sex.** Gene ID, affymetrix probe ID, log ratio and FDR.
(DOCX)

Table S2 **Top 10 differentially expressed genes at baseline between COPD patients and Smokers, stratified by sex.** Gene ID, affymetrix probe ID, log ratio and FDR.
(DOCX)

Table S3 **Top 10 differentially expressed genes at baseline due to gender differences in COPD patients and in healthy smokers.** Gene ID, affymetrix probe ID, log ratio and FDR.
(DOCX)

Data S1 **Detailed methods and additional results.**
(DOCX)
