# Supplementary figures and images for: Systemic Inflammatory Response to Smoking in Chronic Obstructive Pulmonary Disease: Evidence of a Gender Effect
Source: PLoS One. 2014 May 15;9(5):e97491. doi: 10.1371/journal.pone.0097491 (PMC4022517; doi:10.1371/journal.pone.0097491)

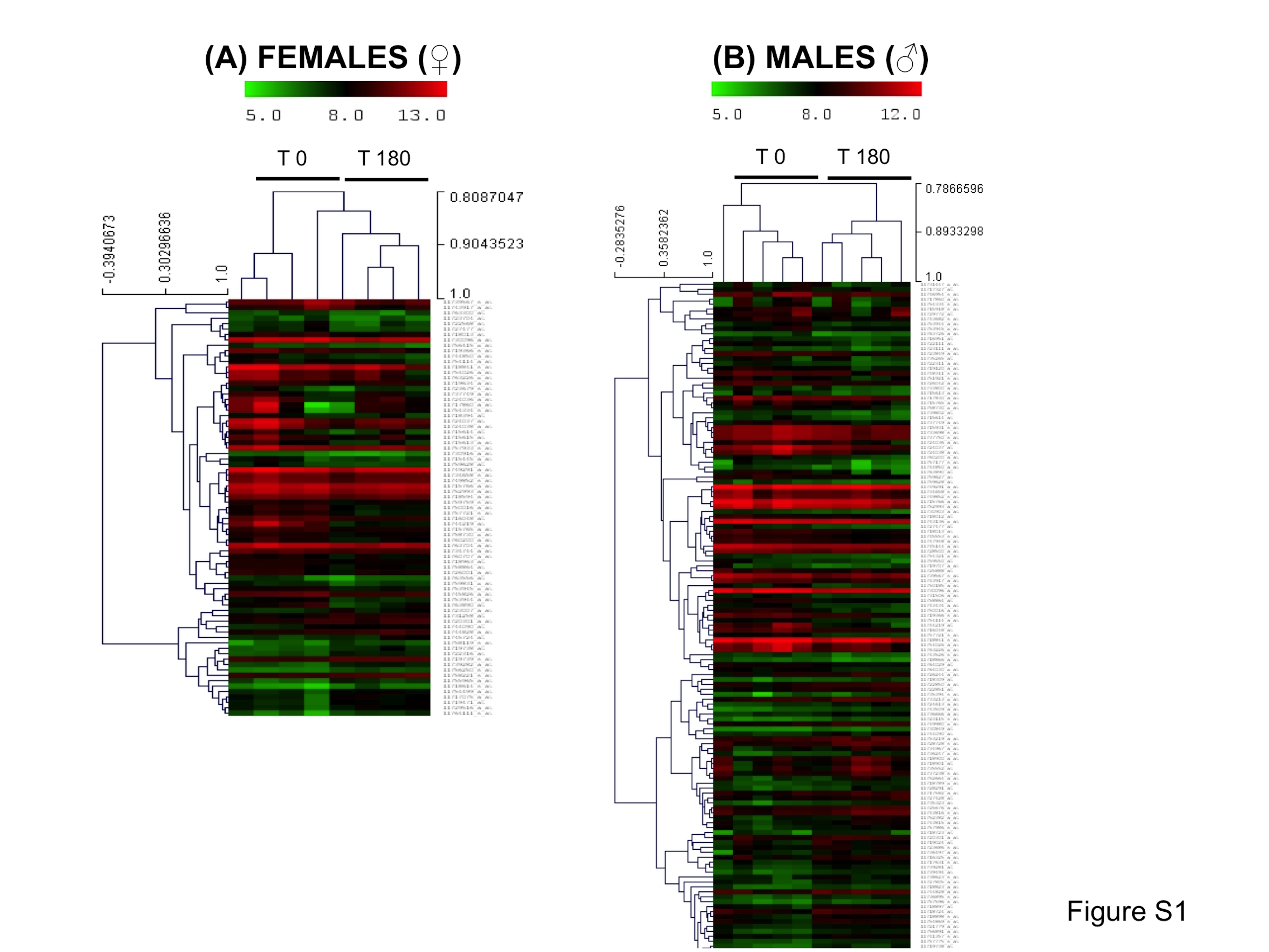

Supplement: Figure S1 — Unsupervised clustering of DE genes in COPD segregated T0 and T180 samples adequately, both in females (n = 57) and males (n = 110) (panels A and B, respectively). (TIFF) [file pone.0097491.s001.tiff]

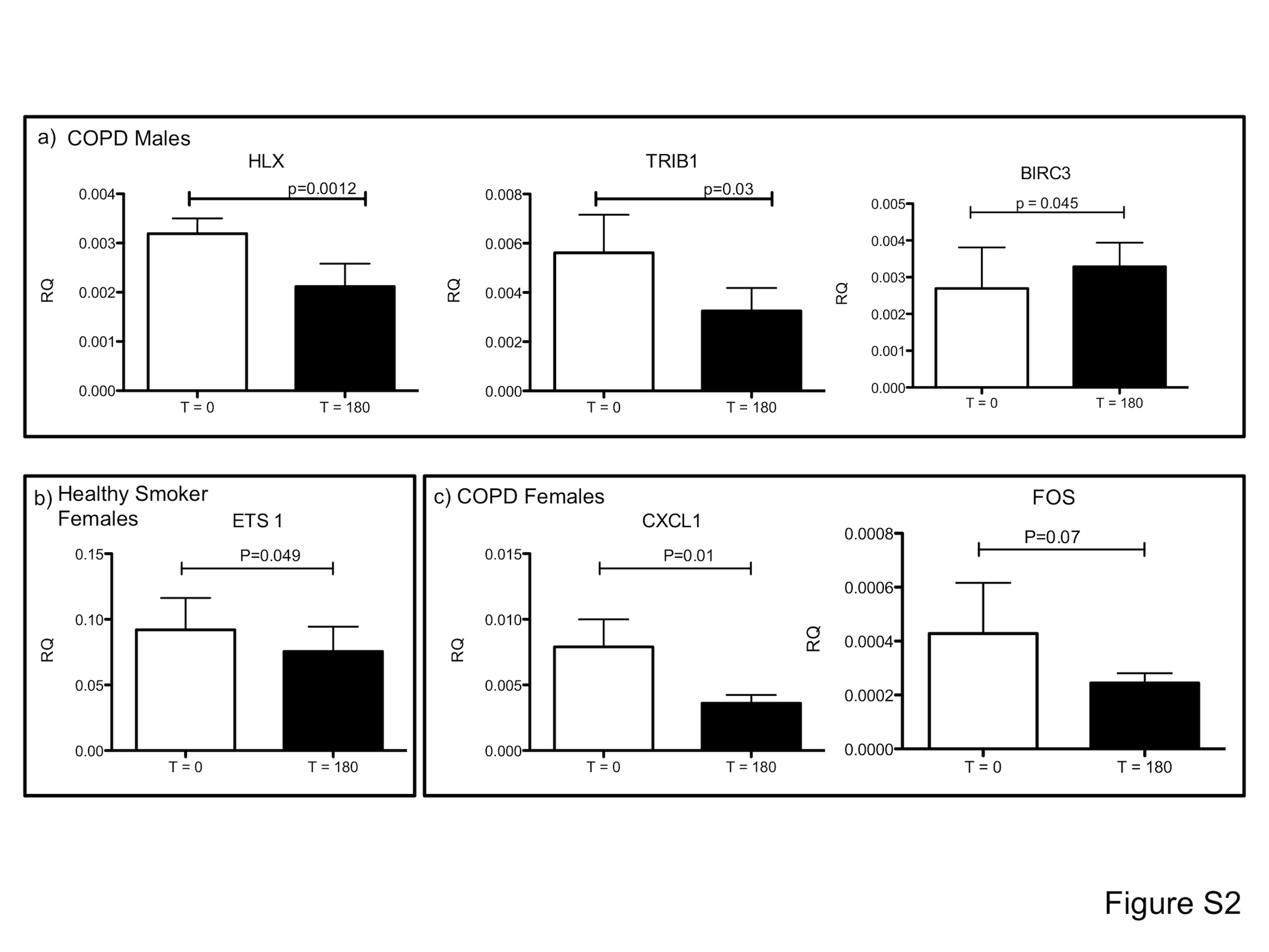

Supplement: Figure S2 — qPCR validation of array results, RQ = relative quantification of target gene mRNA to Actin mRNA calculated using the comparative CT method. Central hub genes in IPA networks were selected for the validation; a) COPD males (HLX, TRIB1 and BIRC3), b) Healthy smoker females (ETS 1) and c) COPD females (CXCL1 and FOS1). (TIFF) [file pone.0097491.s002.tiff]
