# Supplementary material for: Systemic Inflammatory Response to Smoking in Chronic Obstructive Pulmonary Disease: Evidence of a Gender Effect
Source: PLoS One. 2014 May 15;9(5):e97491. doi: 10.1371/journal.pone.0097491 (PMC4022517; doi:10.1371/journal.pone.0097491)
Supplement: Table S1 — Complete list of differentially expressed genes (DE) in COPD patients, smokers (S) and non-smokers (NS) stratified by sex. Gene ID, affymetrix probe ID, log ratio and FDR. (DOCX) [file pone.0097491.s003.docx]

**Table S1**. Complete list of differentially expressed genes (DE) in COPD patients, smokers (S) and non-smokers (NS) stratified by sex. Gene ID, affymetrix probe ID, log ratio and FDR.

| COPD Males | symbol | affyID | Log ratio | FDR |
| --- | --- | --- | --- | --- |
|  | GPR18 | 11732544_a_at | 0.95 | 0.00 |
|  | DCP2 | 11719739_s_at | 0.77 | 0.00 |
|  | DCP2 | 11719738_at | 0.72 | 0.00 |
|  | SPAG9 | 11718897_at | 0.69 | 0.00 |
|  | RBM7 | 11754499_a_at | 0.69 | 0.00 |
|  | ZNF644 | 11729516_a_at | 0.67 | 0.00 |
|  | ZNF644 | 11729261_a_at | 0.66 | 0.00 |
|  | ADRB2 | 11728728_s_at | 0.65 | 0.00 |
|  | ZNF441 | 11735323_at | 0.64 | 0.00 |
|  | ELMOD2 | 11764111_s_at | 0.63 | 0.00 |
|  | BIRC3 | 11722850_a_at | 0.63 | 0.00 |
|  | S1PR1 | 11743816_s_at | 0.62 | 0.00 |
|  | S1PR1 | 11752382_a_at | 0.61 | 0.00 |
|  | MGAT4A | 11739494_at | 0.61 | 0.00 |
|  | CLK1 | 11744828_a_at | 0.60 | 0.00 |
|  | KLHL9 | 11717631_s_at | 0.60 | 0.01 |
|  | ZNF267 | 11754869_s_at | 0.60 | 0.00 |
|  | MGAT4A | 11739493_at | 0.59 | 0.00 |
|  | SLFN5 | 11733213_x_at | 0.59 | 0.01 |
|  | ARRDC3 | 11718723_at | 0.59 | 0.01 |
|  | SOCS4 | 11736895_s_at | 0.59 | 0.00 |
|  | BIRC3 | 11722851_at | 0.59 | 0.00 |
|  | ZNF397 | 11744090_at | 0.59 | 0.00 |
|  | RORA | 11726244_a_at | 0.58 | 0.01 |
|  | ZNF12 | 11756891_a_at | 0.58 | 0.00 |
|  | TRIM23 | 11739281_at | 0.56 | 0.01 |
|  | C10orf18 | 11755774_x_at | 0.56 | 0.00 |
|  | S1PR1 | 11743815_a_at | 0.55 | 0.00 |
|  | ARRDC3 | 11718724_at | 0.55 | 0.00 |
|  | RORA | 11725676_a_at | 0.55 | 0.01 |
|  | KIAA0776 | 11743509_a_at | 0.54 | 0.01 |
|  | NA | 11735394_s_at | 0.54 | 0.01 |
|  | ZNF652 | 11719024_at | 0.53 | 0.01 |
|  | EOMES | 11737238_s_at | 0.53 | 0.00 |
|  | MTX3 | 11723115_s_at | 0.52 | 0.01 |
|  | PTER | 11736666_a_at | 0.52 | 0.01 |
|  | CLK1 | 11716325_a_at | 0.52 | 0.03 |
|  | TNRC6B | 11736497_a_at | 0.51 | 0.04 |
|  | GPR183 | 11727428_at | 0.51 | 0.02 |
|  | KLRF1 | 11753219_a_at | 0.50 | 0.01 |
|  | HOPX | 11749980_x_at | 0.50 | 0.01 |
|  | SH2D1A | 11728291_at | 0.49 | 0.04 |
|  | TGFBR3 | 11718901_at | 0.49 | 0.01 |
|  | FCRL6 | 11735552_at | 0.48 | 0.01 |
|  | FAM46A | 11723686_s_at | 0.47 | 0.03 |
|  | TGFBR3 | 11718900_a_at | 0.47 | 0.03 |
|  | SPAG9 | 11718898_s_at | 0.47 | 0.01 |
|  | DYRK2 | 11724613_a_at | 0.46 | 0.05 |
|  | C6orf120 | 11757775_s_at | 0.46 | 0.04 |
|  | SLA | 11720301_a_at | 0.45 | 0.05 |
|  | C13orf15 | 11717582_a_at | 0.45 | 0.04 |
|  | ACBD5 | 11721779_a_at | 0.45 | 0.02 |
|  | TRIM13 | 11757596_s_at | 0.44 | 0.02 |
|  | FNDC3A | 11741357_s_at | 0.44 | 0.04 |
|  | AMIGO2 | 11736247_x_at | 0.44 | 0.04 |
|  | THEMIS | 11734967_a_at | 0.44 | 0.02 |
|  | MIS12 | 11757986_s_at | 0.44 | 0.03 |
|  | ZNF268 | 11738623_s_at | 0.43 | 0.01 |
|  | CBLL1 | 11734716_at | 0.43 | 0.04 |
|  | S1PR5 | 11752664_a_at | 0.43 | 0.05 |
|  | HEG1 | 11718309_at | 0.43 | 0.05 |
|  | ABHD13 | 11730849_at | 0.42 | 0.04 |
|  | ZNF302 | 11718789_x_at | 0.40 | 0.05 |
|  | UBE2W | 11727805_a_at | 0.39 | 0.05 |
|  | C10orf137 | 11718823_a_at | 0.37 | 0.01 |
|  | G0S2 | 11744219_at | -1.94 | 0.00 |
|  | MXD1 | 11750016_a_at | -1.38 | 0.00 |
|  | PTGS2 | 11724038_a_at | -1.36 | 0.00 |
|  | IL8 | 11754026_a_at | -1.33 | 0.00 |
|  | SNORA28 | 11763890_at | -1.15 | 0.00 |
|  | PTGS2 | 11724037_at | -1.11 | 0.00 |
|  | IL8 | 11763226_x_at | -1.10 | 0.00 |
|  | CSRNP1 | 11757721_s_at | -0.86 | 0.00 |
|  | IL8 | 11718841_s_at | -0.83 | 0.00 |
|  | SAP30 | 11727477_at | -0.79 | 0.00 |
|  | HLX | 11722111_at | -0.79 | 0.00 |
|  | CXCL1 | 11719366_s_at | -0.74 | 0.00 |
|  | PTGS2 | 11724036_a_at | -0.74 | 0.00 |
|  | FKBP5 | 11743917_a_at | -0.73 | 0.00 |
|  | TRIB1 | 11716048_at | -0.72 | 0.00 |
|  | IRS2 | 11718013_at | -0.70 | 0.00 |
|  | SSH2 | 11744850_a_at | -0.70 | 0.00 |
|  | SRP19 | 11758864_at | -0.69 | 0.00 |
|  | CXCL1 | 11754114_a_at | -0.68 | 0.00 |
|  | FKBP5 | 11739567_s_at | -0.66 | 0.00 |
|  | SAT1 | 11760200_a_at | -0.62 | 0.00 |
|  | SMAP2 | 11745553_s_at | -0.62 | 0.00 |
|  | SMAP2 | 11747948_a_at | -0.62 | 0.00 |
|  | BTG2 | 11715613_a_at | -0.61 | 0.00 |
|  | BTG2 | 11715614_at | -0.60 | 0.01 |
|  | CHST11 | 11743434_a_at | -0.59 | 0.00 |
|  | DUSP1 | 11752993_a_at | -0.58 | 0.01 |
|  | FLJ36031 | 11750185_a_at | -0.58 | 0.00 |
|  | IRS2 | 11718012_at | -0.58 | 0.00 |
|  | STK17B | 11726042_a_at | -0.58 | 0.00 |
|  | VCPIP1 | 11739802_at | -0.57 | 0.00 |
|  | CD24 | 11715918_s_at | -0.57 | 0.00 |
|  | SGK1 | 11733698_s_at | -0.56 | 0.01 |
|  | SNORD89 | 11757177_s_at | -0.55 | 0.00 |
|  | TRA2B | 11753944_a_at | -0.53 | 0.00 |
|  | IL1R2 | 11728500_a_at | -0.52 | 0.01 |
|  | SGK1 | 11737750_s_at | -0.52 | 0.01 |
|  | BCL2A1 | 11730096_a_at | -0.52 | 0.00 |
|  | EGR1 | 11754334_s_at | -0.52 | 0.00 |
|  | KYNU | 11719120_a_at | -0.51 | 0.01 |
|  | NCRNA00275 | 11759550_at | -0.51 | 0.01 |
|  | CEBPD | 11764029_at | -0.50 | 0.01 |
|  | JDP2 | 11730903_a_at | -0.50 | 0.01 |
|  | FOS | 11749852_s_at | -0.50 | 0.00 |
|  | SGK1 | 11715931_s_at | -0.50 | 0.02 |
|  | DHRS13 | 11719707_a_at | -0.49 | 0.00 |
|  | CLEC4E | 11745144_a_at | -0.48 | 0.01 |
|  | SGK1 | 11737749_a_at | -0.48 | 0.03 |
|  | MS4A6A | 11723849_a_at | -0.47 | 0.01 |
|  | CEBPD | 11764030_x_at | -0.47 | 0.01 |
|  | NA | 11746954_s_at | -0.47 | 0.03 |
|  | SLC12A9 | 11754321_x_at | -0.47 | 0.01 |
|  | FOS | 11734658_s_at | -0.46 | 0.00 |
|  | DUSP1 | 11758730_x_at | -0.46 | 0.01 |
|  | DUSP1 | 11715766_a_at | -0.46 | 0.02 |
|  | DUSP1 | 11715765_a_at | -0.45 | 0.01 |
|  | RAD23B | 11731506_a_at | -0.44 | 0.01 |
|  | SNCA | 11731447_x_at | -0.44 | 0.02 |
|  | RPL10 | 11763726_a_at | -0.44 | 0.03 |
|  | AHR | 11751921_s_at | -0.44 | 0.05 |
|  | FAM192A | 11718866_a_at | -0.44 | 0.02 |
|  | WIPF1 | 11759628_at | -0.43 | 0.00 |
|  | COQ2 | 11722311_a_at | -0.43 | 0.03 |
|  | SLC31A2 | 11733800_a_at | -0.43 | 0.02 |
|  | CEBPD | 11743136_x_at | -0.41 | 0.05 |
|  | VCAN | 11748311_s_at | -0.41 | 0.05 |
|  | KLF9 | 11717327_at | -0.41 | 0.02 |
|  | TRA2B | 11753945_x_at | -0.40 | 0.03 |
|  | EMILIN2 | 11723111_a_at | -0.40 | 0.04 |
|  | RNASE2 | 11729772_at | -0.40 | 0.04 |
|  | RALBP1 | 11743526_s_at | -0.39 | 0.04 |
|  | USP15 | 11759627_at | -0.39 | 0.04 |
|  | C19orf59 | 11725888_at | -0.39 | 0.04 |
|  | NANS | 11716951_at | -0.38 | 0.04 |
|  | KLF10 | 11743682_s_at | -0.38 | 0.05 |
|  | FOS | 11749291_a_at | -0.38 | 0.01 |
|  | EGR1 | 11717860_a_at | -0.36 | 0.00 |
|  | TSC22D3 | 11717830_a_at | -0.34 | 0.04 |
|  | CLEC4D | 11735265_at | -0.31 | 0.05 |
| COPD Females | symbol | affyID | Log ratio | FDR |
|  | TRIM23 | 11739282_a_at | 0.76 | 0.00 |
|  | DCP2 | 11719738_at | 0.72 | 0.00 |
|  | DCP1A | 11719471_at | 0.72 | 0.01 |
|  | RBM7 | 11754499_a_at | 0.72 | 0.01 |
|  | ELMOD2 | 11764111_s_at | 0.71 | 0.01 |
|  | ZFAND6 | 11717075_a_at | 0.71 | 0.01 |
|  | SNX13 | 11722316_at | 0.70 | 0.00 |
|  | ZNF644 | 11729516_a_at | 0.68 | 0.01 |
|  | PAFAH1B1 | 11755965_a_at | 0.66 | 0.02 |
|  | TMEM170B | 11731258_at | 0.66 | 0.01 |
|  | RABEP1 | 11718614_s_at | 0.62 | 0.01 |
|  | CCPG1 | 11723007_a_at | 0.61 | 0.03 |
|  | TMEM41B | 11758119_s_at | 0.60 | 0.03 |
|  | CLK1 | 11744828_a_at | 0.58 | 0.00 |
|  | DCP2 | 11719739_s_at | 0.57 | 0.03 |
|  | MALAT1 | 11745724_at | 0.53 | 0.01 |
|  | ARRDC3 | 11758221_s_at | 0.53 | 0.02 |
|  | HMGCL | 11756250_s_at | 0.53 | 0.03 |
|  | ZNF397 | 11744090_at | 0.52 | 0.01 |
|  | SLA | 11720301_a_at | 0.49 | 0.02 |
|  | G0S2 | 11744219_at | -2.95 | 0.00 |
|  | PTGS2 | 11724038_a_at | -1.59 | 0.00 |
|  | IL8 | 11763226_x_at | -1.56 | 0.00 |
|  | IL8 | 11754026_a_at | -1.55 | 0.00 |
|  | PTGS2 | 11724037_at | -1.44 | 0.00 |
|  | BTG2 | 11715613_a_at | -1.27 | 0.00 |
|  | MXD1 | 11750016_a_at | -1.19 | 0.00 |
|  | SNORA28 | 11763890_at | -1.17 | 0.00 |
|  | BTG2 | 11715614_at | -1.13 | 0.00 |
|  | IL8 | 11718841_s_at | -1.11 | 0.00 |
|  | PTGS2 | 11724036_a_at | -1.09 | 0.00 |
|  | BTG2 | 11715615_at | -1.09 | 0.00 |
|  | CSRNP1 | 11757721_s_at | -1.06 | 0.00 |
|  | EGR1 | 11717860_a_at | -1.06 | 0.00 |
|  | EGR1 | 11754334_s_at | -1.01 | 0.02 |
|  | TRIB1 | 11716048_at | -1.01 | 0.00 |
|  | CXCL1 | 11719366_s_at | -0.94 | 0.00 |
|  | SAT1 | 11760200_a_at | -0.91 | 0.00 |
|  | BTG2 | 11757933_s_at | -0.79 | 0.00 |
|  | TRA2B | 11753945_x_at | -0.77 | 0.00 |
|  | DUSP1 | 11715765_a_at | -0.74 | 0.00 |
|  | CXCL1 | 11754114_a_at | -0.69 | 0.00 |
|  | FOS | 11734658_s_at | -0.68 | 0.01 |
|  | TRA2B | 11753944_a_at | -0.66 | 0.00 |
|  | DUSP1 | 11758730_x_at | -0.66 | 0.00 |
|  | CCNL1 | 11745826_a_at | -0.65 | 0.00 |
|  | FOS | 11749852_s_at | -0.65 | 0.01 |
|  | SRP19 | 11758864_at | -0.64 | 0.00 |
|  | DUSP1 | 11752993_a_at | -0.62 | 0.01 |
|  | SSH2 | 11744850_a_at | -0.61 | 0.00 |
|  | SAT1 | 11763704_a_at | -0.60 | 0.00 |
|  | NT5C2 | 11722568_at | -0.59 | 0.02 |
|  | SAP30 | 11727477_at | -0.58 | 0.02 |
|  | FOS | 11749291_a_at | -0.56 | 0.02 |
|  | BCL2A1 | 11730096_a_at | -0.56 | 0.01 |
|  | IRS2 | 11718013_at | -0.55 | 0.02 |
|  | RBBP6 | 11730916_a_at | -0.54 | 0.02 |
|  | WIPF1 | 11759628_at | -0.53 | 0.02 |
|  | JUN | 11718394_at | -0.52 | 0.01 |
|  | DNAJB1 | 11715445_a_at | -0.52 | 0.04 |
|  | RBM6 | 11756415_x_at | -0.51 | 0.01 |
|  | FKBP5 | 11743917_a_at | -0.51 | 0.04 |
|  | TRA2A | 11726001_a_at | -0.51 | 0.01 |
|  | MLL5 | 11759831_x_at | -0.50 | 0.04 |
|  | CCNL1 | 11759759_s_at | -0.50 | 0.04 |
|  | ABHD5 | 11723704_at | -0.49 | 0.05 |
|  | EIF4A1 | 11763556_at | -0.49 | 0.02 |
|  | RBM4 | 11763300_at | -0.49 | 0.04 |
|  | FKBP5 | 11739567_s_at | -0.48 | 0.05 |
|  | DUSP1 | 11715766_a_at | -0.48 | 0.04 |
|  | CD69 | 11723679_s_at | -0.47 | 0.05 |
|  | KLF4 | 11719634_a_at | -0.46 | 0.04 |
|  | SGK1 | 11737749_a_at | -0.46 | 0.05 |
|  | CCNL1 | 11718594_a_at | -0.46 | 0.05 |
|  | EIF4A2 | 11760707_a_at | -0.45 | 0.04 |
|  | SON | 11734744_a_at | -0.45 | 0.03 |
|  | NUFIP2 | 11718963_at | -0.43 | 0.04 |
| Smokers Males | symbol | affyID | Log ratio | FDR |
|  | DCP2 | 11719738_at | 0.57 | 0.00 |
|  | DCP2 | 11719739_s_at | 0.55 | 0.00 |
|  | SLC30A1 | 11758948_s_at | 0.53 | 0.01 |
|  | ABCG1 | 11725515_a_at | 0.49 | 0.04 |
|  | IL18R1 | 11735275_at | 0.48 | 0.05 |
|  | FGFBP2 | 11750086_s_at | 0.48 | 0.03 |
|  | XYLT1 | 11727221_at | 0.45 | 0.03 |
|  | ZNF564 | 11727148_s_at | 0.43 | 0.04 |
|  | ADRB2 | 11728728_s_at | 0.42 | 0.05 |
|  | ADH5 | 11716392_s_at | 0.38 | 0.05 |
|  | NA | 11763447_x_at | 0.36 | 0.04 |
|  | SIPA1L2 | 11744185_a_at | 0.36 | 0.04 |
|  | IL8 | 11754026_a_at | -1.29 | 0.00 |
|  | G0S2 | 11744219_at | -1.26 | 0.00 |
|  | PTGS2 | 11724038_a_at | -1.13 | 0.00 |
|  | IL8 | 11763226_x_at | -1.10 | 0.00 |
|  | PTGS2 | 11724037_at | -1.06 | 0.00 |
|  | SNORA28 | 11763890_at | -1.04 | 0.00 |
|  | MXD1 | 11750016_a_at | -0.98 | 0.00 |
|  | PTGS2 | 11724036_a_at | -0.85 | 0.00 |
|  | DUSP1 | 11715765_a_at | -0.77 | 0.00 |
|  | BTG2 | 11715613_a_at | -0.74 | 0.00 |
|  | BTG2 | 11715615_at | -0.73 | 0.00 |
|  | IL8 | 11718841_s_at | -0.72 | 0.00 |
|  | DUSP1 | 11758730_x_at | -0.72 | 0.00 |
|  | HSPH1 | 11717096_a_at | -0.69 | 0.00 |
|  | SRP19 | 11758864_at | -0.64 | 0.00 |
|  | BTG2 | 11715614_at | -0.62 | 0.00 |
|  | CD69 | 11723679_s_at | -0.61 | 0.00 |
|  | CXCL1 | 11754114_a_at | -0.60 | 0.02 |
|  | CXCL1 | 11719366_s_at | -0.60 | 0.03 |
|  | HSPH1 | 11755205_a_at | -0.58 | 0.00 |
|  | DUSP1 | 11752993_a_at | -0.55 | 0.01 |
|  | CPSF6 | 11746369_a_at | -0.52 | 0.02 |
|  | TRA2B | 11753945_x_at | -0.51 | 0.02 |
|  | CREB5 | 11762865_a_at | -0.50 | 0.00 |
|  | CLC | 11727473_at | -0.50 | 0.02 |
|  | FOS | 11749852_s_at | -0.48 | 0.01 |
|  | CSRNP1 | 11757721_s_at | -0.47 | 0.02 |
|  | NA | 11742059_a_at | -0.47 | 0.03 |
|  | FOS | 11734658_s_at | -0.47 | 0.02 |
|  | HSPH1 | 11749659_s_at | -0.46 | 0.03 |
|  | DDX17 | 11715474_at | -0.46 | 0.03 |
|  | MLL5 | 11759831_x_at | -0.45 | 0.04 |
|  | ETS1 | 11752387_a_at | -0.44 | 0.01 |
|  | DDX5 | 11733798_at | -0.43 | 0.04 |
|  | SMAP2 | 11747948_a_at | -0.42 | 0.00 |
|  | NT5C2 | 11722568_at | -0.42 | 0.01 |
|  | SSH2 | 11744850_a_at | -0.41 | 0.02 |
|  | SRSF10 | 11721310_s_at | -0.41 | 0.05 |
|  | SAP30 | 11727477_at | -0.41 | 0.01 |
|  | HSPH1 | 11755206_x_at | -0.40 | 0.05 |
|  | FKBP5 | 11739567_s_at | -0.40 | 0.00 |
|  | TSC22D3 | 11717830_a_at | -0.40 | 0.01 |
|  | POU2AF1 | 11726153_at | -0.39 | 0.02 |
|  | SP100 | 11744673_a_at | -0.37 | 0.05 |
|  | TSC22D3 | 11751415_a_at | -0.36 | 0.01 |
|  | CEP350 | 11719054_at | -0.36 | 0.05 |
|  | IRS2 | 11718013_at | -0.33 | 0.00 |
|  | FKBP5 | 11743917_a_at | -0.31 | 0.00 |
|  | SMAP2 | 11745553_s_at | -0.31 | 0.02 |
|  | SLC31A2 | 11748338_a_at | -0.30 | 0.04 |
|  | IRS2 | 11718012_at | -0.30 | 0.04 |
|  | SMAP2 | 11719845_a_at | -0.29 | 0.01 |
|  | DDIT4 | 11743972_a_at | -0.28 | 0.03 |
|  | BAX | 11759444_x_at | -0.28 | 0.03 |
|  | EGR1 | 11717860_a_at | -0.08 | 0.05 |
| Smokers females | symbol | affyID | Log ratio | FDR |
|  | ARRDC3 | 11758221_s_at | 0.77 | 0.00 |
|  | REPS2 | 11722348_a_at | 0.71 | 0.00 |
|  | RBM7 | 11754499_a_at | 0.69 | 0.00 |
|  | ARRDC3 | 11718724_at | 0.68 | 0.00 |
|  | DCP2 | 11719738_at | 0.64 | 0.00 |
|  | FOSL2 | 11763171_at | 0.63 | 0.00 |
|  | DCP2 | 11719739_s_at | 0.62 | 0.00 |
|  | SPAG9 | 11718898_s_at | 0.62 | 0.00 |
|  | CDA | 11753626_a_at | 0.61 | 0.00 |
|  | NA | 11764259_at | 0.61 | 0.00 |
|  | CPEB2 | 11724951_s_at | 0.60 | 0.00 |
|  | PKN2 | 11724840_at | 0.59 | 0.00 |
|  | ZNF267 | 11754869_s_at | 0.58 | 0.00 |
|  | TNFSF14 | 11741290_a_at | 0.57 | 0.00 |
|  | DHRS9 | 11723899_a_at | 0.57 | 0.00 |
|  | TBX21 | 11732538_at | 0.57 | 0.00 |
|  | ZNF441 | 11735323_at | 0.56 | 0.00 |
|  | ABCG1 | 11725516_s_at | 0.55 | 0.00 |
|  | NA | 11735394_s_at | 0.55 | 0.00 |
|  | TNFSF14 | 11738883_x_at | 0.53 | 0.00 |
|  | TMCC3 | 11728839_s_at | 0.53 | 0.01 |
|  | ARRDC3 | 11718723_at | 0.53 | 0.00 |
|  | GPR18 | 11732544_a_at | 0.52 | 0.00 |
|  | MMP9 | 11719657_a_at | 0.51 | 0.00 |
|  | TRIM23 | 11739282_a_at | 0.50 | 0.02 |
|  | C20orf3 | 11717151_s_at | 0.49 | 0.01 |
|  | NA | 11763447_x_at | 0.49 | 0.02 |
|  | MGAM | 11731270_a_at | 0.48 | 0.02 |
|  | FADD | 11716800_at | 0.47 | 0.02 |
|  | GPR56 | 11716395_a_at | 0.47 | 0.02 |
|  | NA | 11754267_x_at | 0.47 | 0.02 |
|  | CPD | 11727131_at | 0.47 | 0.02 |
|  | C13orf18 | 11755405_a_at | 0.47 | 0.02 |
|  | KCNJ15 | 11732454_at | 0.47 | 0.02 |
|  | ING1 | 11727958_a_at | 0.47 | 0.02 |
|  | XYLT1 | 11727221_at | 0.46 | 0.02 |
|  | LRRC57 | 11754389_x_at | 0.46 | 0.02 |
|  | SLC30A1 | 11758948_s_at | 0.46 | 0.02 |
|  | C13orf18 | 11754859_x_at | 0.46 | 0.02 |
|  | S1PR5 | 11752664_a_at | 0.46 | 0.02 |
|  | SLC16A6 | 11754629_s_at | 0.46 | 0.03 |
|  | RRM2B | 11736193_a_at | 0.45 | 0.03 |
|  | ZNF644 | 11729261_a_at | 0.45 | 0.02 |
|  | MME | 11755860_a_at | 0.45 | 0.02 |
|  | TM2D3 | 11746436_a_at | 0.44 | 0.03 |
|  | CST7 | 11725124_at | 0.44 | 0.03 |
|  | FBXO28 | 11740787_a_at | 0.44 | 0.03 |
|  | TRIM23 | 11739281_at | 0.43 | 0.04 |
|  | SPAG9 | 11718897_at | 0.43 | 0.04 |
|  | MAD2L1BP | 11716880_s_at | 0.42 | 0.02 |
|  | SLA | 11720301_a_at | 0.42 | 0.04 |
|  | C13orf18 | 11734852_a_at | 0.42 | 0.04 |
|  | JHDM1D | 11755223_a_at | 0.42 | 0.04 |
|  | NA | 11760097_s_at | 0.41 | 0.05 |
|  | MME | 11747693_a_at | 0.41 | 0.04 |
|  | C20orf177 | 11759407_at | 0.41 | 0.04 |
|  | TGFBR3 | 11718901_at | 0.41 | 0.03 |
|  | ACBD5 | 11721779_a_at | 0.40 | 0.04 |
|  | ANXA3 | 11724283_a_at | 0.40 | 0.04 |
|  | PADI4 | 11729853_at | 0.39 | 0.04 |
|  | ABCG1 | 11725517_x_at | 0.39 | 0.05 |
|  | MOSC1 | 11731778_a_at | 0.39 | 0.03 |
|  | ADRB2 | 11728728_s_at | 0.39 | 0.04 |
|  | CHST11 | 11759496_at | 0.37 | 0.05 |
|  | VNN2 | 11746356_a_at | 0.37 | 0.04 |
|  | IL18RAP | 11732017_a_at | 0.36 | 0.03 |
|  | CLEC4D | 11735265_at | 0.36 | 0.02 |
|  | WIPI2 | 11734801_a_at | 0.34 | 0.04 |
|  | LOC254896 | 11760084_x_at | 0.22 | 0.04 |
|  | G0S2 | 11744219_at | -1.60 | 0.00 |
|  | MXD1 | 11750016_a_at | -1.23 | 0.00 |
|  | SNORA28 | 11763890_at | -1.42 | 0.00 |
|  | PTGS2 | 11724038_a_at | -0.98 | 0.00 |
|  | PTGS2 | 11724037_at | -0.90 | 0.00 |
|  | CSRNP1 | 11757721_s_at | -0.89 | 0.00 |
|  | IL8 | 11754026_a_at | -0.78 | 0.00 |
|  | IL8 | 11763226_x_at | -0.77 | 0.00 |
|  | DDIT4 | 11743972_a_at | -0.77 | 0.00 |
|  | SAT1 | 11760200_a_at | -0.74 | 0.00 |
|  | KLF9 | 11717326_at | -0.72 | 0.00 |
|  | TRA2B | 11753945_x_at | -0.71 | 0.00 |
|  | SSH2 | 11744850_a_at | -0.66 | 0.00 |
|  | DUSP1 | 11715765_a_at | -0.65 | 0.01 |
|  | SRP19 | 11758864_at | -0.64 | 0.00 |
|  | TSC22D3 | 11717830_a_at | -0.62 | 0.00 |
|  | ETS1 | 11752387_a_at | -0.61 | 0.00 |
|  | EIF4A1 | 11762280_a_at | -0.60 | 0.00 |
|  | PTGS2 | 11724036_a_at | -0.59 | 0.02 |
|  | KLF9 | 11717327_at | -0.56 | 0.02 |
|  | DUSP1 | 11758730_x_at | -0.54 | 0.03 |
|  | PPIP5K2 | 11726148_a_at | -0.53 | 0.01 |
|  | TRA2B | 11753944_a_at | -0.53 | 0.02 |
|  | TRIB1 | 11716048_at | -0.53 | 0.02 |
|  | KLF10 | 11743682_s_at | -0.52 | 0.02 |
|  | FKBP5 | 11743917_a_at | -0.52 | 0.01 |
|  | TLE4 | 11719997_a_at | -0.51 | 0.02 |
|  | NA | 11746954_s_at | -0.51 | 0.02 |
|  | EIF4A2 | 11760707_a_at | -0.51 | 0.02 |
|  | SMAP2 | 11719845_a_at | -0.50 | 0.01 |
|  | KLF10 | 11733068_s_at | -0.49 | 0.03 |
|  | PABPN1 | 11763165_at | -0.49 | 0.02 |
|  | NA | 11740038_s_at | -0.48 | 0.02 |
|  | SMAP2 | 11747948_a_at | -0.48 | 0.03 |
|  | IL6ST | 11759038_at | -0.48 | 0.04 |
|  | IRS2 | 11718013_at | -0.47 | 0.03 |
|  | HLX | 11722111_at | -0.46 | 0.01 |
|  | EIF2D | 11732960_a_at | -0.46 | 0.04 |
|  | SAP30 | 11727477_at | -0.45 | 0.02 |
|  | FKBP5 | 11739567_s_at | -0.44 | 0.01 |
|  | TSC22D3 | 11751415_a_at | -0.44 | 0.03 |
|  | CYBB | 11751821_a_at | -0.44 | 0.04 |
|  | POU2AF1 | 11726153_at | -0.42 | 0.04 |
|  | NSUN6 | 11730890_at | -0.39 | 0.05 |
|  | LILRB4 | 11730813_at | -0.35 | 0.04 |
|  | BCL2 | 11721376_at | -0.47 | 0.04 |
|  | ARGLU1 | 11719931_x_at | -0.48 | 0.03 |
|  | CCNL1 | 11745826_a_at | -0.49 | 0.02 |
|  | AHR | 11751921_s_at | -0.52 | 0.01 |
|  | BTG2 | 11715615_at | -0.54 | 0.03 |
|  | ANKRD10 | 11761390_a_at | -0.60 | 0.00 |
|  | CCNL1 | 11759759_s_at | -0.60 | 0.00 |
|  | CD69 | 11723679_s_at | -0.60 | 0.01 |
| Non-smokers males | symbol | affyID | Log ratio | FDR |
|  | DCP2 | 11719738_at | 0.84 | 0.00 |
|  | ABCG1 | 11725516_s_at | 0.66 | 0.00 |
|  | RBM7 | 11754499_a_at | 0.61 | 0.00 |
|  | GPR18 | 11732544_a_at | 0.58 | 0.00 |
|  | PDE3B | 11730473_at | 0.56 | 0.01 |
|  | SLC30A1 | 11758948_s_at | 0.54 | 0.01 |
|  | ZNF267 | 11754869_s_at | 0.51 | 0.02 |
|  | ZNF644 | 11729516_a_at | 0.47 | 0.02 |
|  | C13orf18 | 11754823_a_at | 0.47 | 0.02 |
|  | ELMOD2 | 11764111_s_at | 0.44 | 0.03 |
|  | PREPL | 11718393_s_at | 0.38 | 0.03 |
|  | EGR1 | 11717860_a_at | 0.22 | 0.01 |
|  | SNORA28 | 11763890_at | -1.68 | 0.00 |
|  | G0S2 | 11744219_at | -1.27 | 0.00 |
|  | MXD1 | 11750016_a_at | -1.22 | 0.00 |
|  | DUSP1 | 11715765_a_at | -1.13 | 0.00 |
|  | PTGS2 | 11724038_a_at | -1.11 | 0.00 |
|  | FOS | 11734658_s_at | -1.08 | 0.00 |
|  | IL8 | 11754026_a_at | -1.03 | 0.00 |
|  | NCRNA00275 | 11759550_at | -0.89 | 0.00 |
|  | DDIT4 | 11743972_a_at | -0.79 | 0.00 |
|  | ELF2 | 11760507_at | -0.70 | 0.00 |
|  | BTG2 | 11715613_a_at | -0.65 | 0.00 |
|  | SNORD89 | 11757177_s_at | -0.64 | 0.00 |
|  | NT5C2 | 11722568_at | -0.63 | 0.00 |
|  | SSH2 | 11744850_a_at | -0.62 | 0.00 |
|  | CXCL1 | 11754114_a_at | -0.62 | 0.00 |
|  | FKBP5 | 11743917_a_at | -0.62 | 0.00 |
|  | RBM4 | 11763300_at | -0.62 | 0.00 |
|  | IRS2 | 11718013_at | -0.61 | 0.00 |
|  | PABPN1 | 11763165_at | -0.59 | 0.00 |
|  | PION | 11755043_a_at | -0.59 | 0.00 |
|  | STK4 | 11760475_at | -0.57 | 0.00 |
|  | CCNL1 | 11745826_a_at | -0.56 | 0.00 |
|  | CREB5 | 11762865_a_at | -0.54 | 0.03 |
|  | EIF4A2 | 11760707_a_at | -0.54 | 0.01 |
|  | TSC22D3 | 11717830_a_at | -0.54 | 0.01 |
|  | PXN | 11760316_x_at | -0.53 | 0.01 |
|  | VCPIP1 | 11739802_at | -0.53 | 0.01 |
|  | CD69 | 11723679_s_at | -0.51 | 0.02 |
|  | TLE4 | 11719997_a_at | -0.51 | 0.01 |
|  | SAP30 | 11727477_at | -0.51 | 0.01 |
|  | RUNX3 | 11743428_a_at | -0.50 | 0.01 |
|  | TXK | 11759347_x_at | -0.49 | 0.03 |
|  | MLL5 | 11759830_at | -0.49 | 0.02 |
|  | MYBL1 | 11725861_a_at | -0.48 | 0.02 |
|  | KLRD1 | 11753484_x_at | -0.48 | 0.02 |
|  | KLF9 | 11717326_at | -0.48 | 0.02 |
|  | GATAD1 | 11725120_a_at | -0.48 | 0.02 |
|  | BAZ1B | 11716388_a_at | -0.47 | 0.01 |
|  | ETS1 | 11752387_a_at | -0.46 | 0.03 |
|  | MAPK14 | 11759946_at | -0.46 | 0.01 |
|  | GZMB | 11724900_a_at | -0.45 | 0.03 |
|  | FNBP4 | 11717944_at | -0.45 | 0.03 |
|  | SPON2 | 11754601_s_at | -0.45 | 0.03 |
|  | MLL3 | 11739411_a_at | -0.45 | 0.03 |
|  | NFAT5 | 11717466_a_at | -0.45 | 0.04 |
|  | CSRNP1 | 11757721_s_at | -0.44 | 0.02 |
|  | SAT1 | 11760200_a_at | -0.43 | 0.05 |
|  | ABHD5 | 11723704_at | -0.42 | 0.01 |
|  | GON4L | 11723152_a_at | -0.42 | 0.04 |
|  | SMAP2 | 11719845_a_at | -0.40 | 0.01 |
|  | UBA6 | 11730893_a_at | -0.38 | 0.04 |
| Non-smokers females | symbol | affyID | Log ratio | FDR |
|  | EGR1 | 11717860_a_at | 0.95 | 0.00 |
|  | EGR1 | 11754334_s_at | 0.66 | 0.00 |
|  | DIS3L | 11744310_x_at | 0.59 | 0.01 |
|  | HINT3 | 11725674_at | 0.52 | 0.02 |
|  | TRIM23 | 11739281_at | 0.45 | 0.00 |
|  | RBM7 | 11754499_a_at | 0.35 | 0.01 |
|  | MGAT4A | 11739493_at | 0.35 | 0.03 |
|  | SLC16A6 | 11754629_s_at | 0.34 | 0.03 |
|  | DCP1A | 11719471_at | 0.32 | 0.03 |
|  | GPR18 | 11732544_a_at | 0.30 | 0.03 |
|  | SPAG9 | 11718898_s_at | 0.29 | 0.02 |
|  | DCP2 | 11719739_s_at | 0.28 | 0.01 |
|  | DCP2 | 11719738_at | 0.28 | 0.00 |
|  | ZNF441 | 11735323_at | 0.26 | 0.01 |
|  | MTX3 | 11723115_s_at | 0.19 | 0.01 |
|  | AP3B1 | 11719295_a_at | 0.12 | 0.00 |
|  | RIF1 | 11731684_at | 0.09 | 0.01 |
|  | PTGS1 | 11722209_a_at | -0.67 | 0.01 |
|  | TM9SF3 | 11751615_a_at | -0.67 | 0.03 |
|  | G0S2 | 11744219_at | -0.58 | 0.00 |
|  | PTGS2 | 11724038_a_at | -0.58 | 0.00 |
|  | MXD1 | 11750016_a_at | -0.51 | 0.00 |
|  | HLX | 11722111_at | -0.49 | 0.04 |
|  | PTGS2 | 11724037_at | -0.46 | 0.01 |
|  | FKBP5 | 11739567_s_at | -0.40 | 0.00 |
|  | FKBP5 | 11743917_a_at | -0.37 | 0.00 |
|  | IL8 | 11754026_a_at | -0.37 | 0.03 |
|  | ARHGEF40 | 11755297_a_at | -0.36 | 0.03 |
|  | RELL1 | 11726839_s_at | -0.35 | 0.05 |
|  | AK2 | 11733317_s_at | -0.34 | 0.03 |
|  | IL8 | 11763226_x_at | -0.34 | 0.03 |
|  | SMAP2 | 11719845_a_at | -0.31 | 0.01 |
|  | CCDC85B | 11721483_at | -0.31 | 0.03 |
|  | TSC22D3 | 11751415_a_at | -0.31 | 0.00 |
|  | CLEC4E | 11745144_a_at | -0.31 | 0.04 |
|  | SMAP2 | 11747948_a_at | -0.31 | 0.01 |
|  | NELL2 | 11740910_a_at | -0.27 | 0.03 |
|  | HSPA5 | 11725934_x_at | -0.27 | 0.05 |
|  | TSC22D3 | 11717830_a_at | -0.26 | 0.01 |
|  | SMAP2 | 11745553_s_at | -0.25 | 0.01 |
|  | SNORA28 | 11763890_at | -0.24 | 0.01 |
|  | SLC31A2 | 11733800_a_at | -0.19 | 0.01 |
|  | HSPH1 | 11755205_a_at | -0.18 | 0.02 |
|  | SSH2 | 11744850_a_at | -0.04 | 0.03 |
