# Supplementary material for: Systemic Inflammatory Response to Smoking in Chronic Obstructive Pulmonary Disease: Evidence of a Gender Effect
Source: PLoS One. 2014 May 15;9(5):e97491. doi: 10.1371/journal.pone.0097491 (PMC4022517; doi:10.1371/journal.pone.0097491)
Supplement: Table S2 — Top 10 differentially expressed genes at baseline between COPD patients and Smokers, stratified by sex. Gene ID, affymetrix probe ID, log ratio and FDR. (DOCX) [file pone.0097491.s004.docx]

**Table S2**. Top 10 differentially expressed genes at baseline between COPD patients and Smokers, stratified by sex. Gene ID, affymetrix probe ID, log ratio and FDR.

| Females TOP 10 genes COPD vs. Smokers | | | | | |
| --- | --- | --- | --- | --- | --- |
|  |  | symbol | affyID | Log ratio | FDR |
| Up-regulated genes | 1 | EPSTI1 | 11731181_a_at | 1.68 | 0 |
|  | 2 | SIGLEC5 | 11729739_at | 1.65 | 0 |
|  | 3 | SAMD9L | 11724117_x_at | 1.63 | 0 |
|  | 4 | LCN2 | 11757634_a_at | 1.54 | 0 |
|  | 5 | C19orf59 | 11725888_at | 1.51 | 0 |
|  | 6 | PARP9 | 11756306_a_at | 1.5 | 0 |
|  | 7 | FCGR1A | 11736311_x_at | 1.49 | 0 |
|  | 8 | ANXA3 | 11756334_x_at | 1.43 | 0 |
|  | 9 | ALPL | 11729337_x_at | 1.36 | 0 |
|  | 10 | DYSF | 11747936_a_at | 1.35 | 0 |
| Down-regulated genes | 1 | CCR3 | 11741322_a_at | -1.41 | 0 |
|  | 2 | HLA-DPB1 | 11760878_x_at | -1.38 | 0 |
|  | 3 | MS4A1 | 11755858_a_at | -1.37 | 0 |
|  | 4 | HLA-DQA1 | 11753898_x_at | -1.28 | 0 |
|  | 5 | IGJ | 11735990_x_at | -1.27 | 0 |
|  | 6 | CLC | 11727473_at | -1.25 | 0 |
|  | 7 | FCER1A | 11728532_at | -1.23 | 0 |
|  | 8 | FAIM3 | 11721815_a_at | -1.18 | 0 |
|  | 9 | C8orf59 | 11751867_a_at | -1.17 | 0 |
|  | 10 | BANK1 | 11726203_a_at | -1.14 | 0 |
| Males TOP 10 genes COPD vs. Smokers | | | | | |
|  |  | symbol | affyID | Log ratio | FDR |
| Up-regulated genes | 1 | HLA-DPB1 | 11757801_x_at | 1.62 | 0 |
|  | 2 | CHURC1 | 11721831_at | 0.97 | 0 |
|  | 3 | FGFBP2 | 11750086_s_at | 0.93 | 0 |
|  | 4 | CHURC1 | 11721832_s_at | 0.87 | 0 |
|  | 5 | ZNF429 | 11740148_x_at | 0.85 | 0 |
|  | 6 | FGFBP2 | 11722782_at | 0.73 | 0 |
|  | 7 | IL18RAP | 11732017_a_at | 0.72 | 0 |
|  | 8 | GPR56 | 11716395_a_at | 0.7 | 0 |
|  | 9 | ZNF429 | 11750315_x_at | 0.67 | 0 |
|  | 10 | PDK4 | 11716974_a_at | 0.57 | 0 |
| Down-regulated genes | 1 | PI3 | 11721650_at | -1.4 | 0 |
|  | 2 | MUTED | 11715395_a_at | -1.28 | 0 |
|  | 3 | PF4V1 | 11738595_at | -1.19 | 0 |
|  | 4 | HLA-DQA1 | 11753898_x_at | -1.1 | 0 |
|  | 5 | PF4V1 | 11738596_x_at | -0.85 | 0 |
|  | 6 | IGJ | 11735990_x_at | -0.74 | 0 |
|  | 7 | IFITM3 | 11715239_x_at | -0.73 | 0 |
|  | 8 | NRGN | 11718787_at | -0.69 | 0 |
|  | 9 | FAM65B | 11747281_a_at | -0.69 | 0.0001 |
|  | 10 | SPARC | 11715604_x_at | -0.68 | 0 |
