# Supplementary material for: Systemic Inflammatory Response to Smoking in Chronic Obstructive Pulmonary Disease: Evidence of a Gender Effect
Source: PLoS One. 2014 May 15;9(5):e97491. doi: 10.1371/journal.pone.0097491 (PMC4022517; doi:10.1371/journal.pone.0097491)
Supplement: Table S3 — Top 10 differentially expressed genes at baseline due to gender differences in COPD patients and in healthy smokers. Gene ID, affymetrix probe ID, log ratio and FDR. (DOCX) [file pone.0097491.s005.docx]

**Table S3**. Top 10 differentially expressed genes at baseline due to gender differences in COPD patients and in healthy smokers. Gene ID, affymetrix probe ID, log ratio and FDR.

| TOP 10 genes different at baseline in COPD males vs. COPD females | | | | | |
| --- | --- | --- | --- | --- | --- |
| Up-regulated genes |  | symbol | affyID | Log ratio | FDR |
|  | 1 | PTPRN2 | 11729976_a_at | 1.04 | 0 |
|  | 2 | CFD | 11725980_at | 0.89 | 0 |
| Down-regulated genes | 1 | SAMD9L | 11724117_x_at | -1.73 | 0 |
|  | 2 | FCGR1A | 11736311_x_at | -1.72 | 0 |
|  | 3 | PARP9 | 11756306_a_at | -1.52 | 0 |
|  | 4 | EPSTI1 | 11731181_a_at | -1.46 | 0 |
|  | 5 | GBP5 | 11733439_a_at | -1.45 | 0 |
|  | 6 | AIM2 | 11730457_a_at | -1.41 | 0 |
|  | 7 | IFITM3 | 11715239_x_at | -1.37 | 0 |
|  | 8 | P2RY14 | 11733632_s_at | -1.36 | 0 |
|  | 9 | MAPK14 | 11759946_at | -1.34 | 0 |
|  | 10 | NAIP | 11736898_s_at | -1.3 | 0 |
| TOP 10 genes different at baseline in Smoker males vs. smokers females | | | | | |
|  |  | symbol | affyID | Log ratio | FDR |
| Up-regulated genes | 1 | LCN2 | 11757634_a_at | 1.13 | 0 |
|  | 2 | CAMP | 11726688_at | 1.07 | 0 |
|  | 3 | SIGLEC5 | 11729739_at | 1.03 | 0 |
|  | 4 | PTPRN2 | 11729976_a_at | 0.95 | 0 |
|  | 5 | FKBP1A | 11753825_x_at | 0.84 | 0 |
|  | 6 | C1orf183 | 11734941_x_at | 0.83 | 0 |
|  | 7 | SLPI | 11716033_at | 0.82 | 0 |
|  | 8 | DHRS9 | 11723899_a_at | 0.79 | 0 |
|  | 9 | SLC45A4 | 11759568_at | 0.78 | 0 |
|  | 10 | AGPAT9 | 11725496_a_at | 0.76 | 0 |
| Down-regulated genes | 1 | CHURC1 | 11721832_s_at | -1.1 | 0 |
|  | 2 | HLA-DPB1 | 11757801_x_at | -1.08 | 0 |
|  | 3 | MXRA7 | 11758622_s_at | -1.02 | 0 |
|  | 4 | CLEC12A | 11762101_at | -0.99 | 0 |
|  | 5 | EIF1AX | 11716779_at | -0.87 | 0 |
|  | 6 | sep-06 | 11721626_a_at | -0.86 | 0 |
|  | 7 | ALG13 | 11763168_s_at | -0.77 | 0 |
|  | 8 | TCL1A | 11723206_at | -0.75 | 0 |
|  | 9 | RPS26 | 11757761_x_at | -0.71 | 0 |
|  | 10 | TNFRSF25 | 11722134_a_at | -0.69 | 0 |
