# Supplementary material for: Systemic Inflammatory Response to Smoking in Chronic Obstructive Pulmonary Disease: Evidence of a Gender Effect
Source: PLoS One. 2014 May 15;9(5):e97491. doi: 10.1371/journal.pone.0097491 (PMC4022517; doi:10.1371/journal.pone.0097491)
Supplement: Data S1 — Detailed methods and additional results. (DOCX) [file pone.0097491.s006.docx]

Friday, 25 April 2014

**Supporting information**

**Systemic inflammatory response to smoking in Chronic Obstructive Pulmonary Disease: Evidence of a gender effect**

Rosa Faner* ^1,2,3^, Nuria Gonzalez* ^1^, Tamara Cruz ^1,2^, Susana Graciela Kalko ^4^, Alvar Agustí ^1,2,3,5,6^

**METHODS**

**Participants**

We included in the study 30 volunteers recruited from the outpatient clinic of our Institution and public advertisement. All COPD patients were clinically stable and were treated with long-acting bronchodilators if needed. None of them was using inhaled or oral steroids. Participants with bronchiectasis, cancer, any chronic inflammatory disease and/or receiving treatment any anti-inflammatory drugs were excluded from the study.

**Blood sampling and processing**

A cannula (BD Preset, Eclipse, BD Diagnostics, New Jersey, US) was inserted into an ante-cubital vein to allow serial blood sampling (10 ml each. Plasma was aliquoted and stored at -70ºC until analysis whereas the buffy coat was immediately used for the measurements of cellular, protein and transcriptomics markers of inflammation.

**Measurements**

*Peripheral blood lymphocyte subpopulations*

50μl of peripheral blood were stained with anti CD3-Fitc/CD16+CD56+PE simultest, anti-CD19-PE-Cy5, anti-CD8-PE-Cy7and CD4-APC, all purchased from BD Biosciences (USA) during 30 minutes at room temperature. Then, red blood cells were lysed with 1ml of lysis buffer, (BD FACS Lysing Solution, USA), samples washed with PBS and images acquired in a FacsCanto (BD Biosciences, USA). Ten thousand lymphocytes were gated and analyzed. Natural killer cells were defined as CD3-, CD16+ CD56+; B cells as CD19+, T cells were divided in CD3+ and CD8+ or CD3+ and CD4+.

*Leukocyte mRNA profile*

RNA samples were hybridized to Affymetrix HG-U219 arrays following Affymetrix’s protocols. In brief, from 150 ng of total RNA a biotin labeled cRNA was generated by reverse transcription followed by *in vitro* transcription (IVT). After cRNA fragmentation, samples were hybridized on a GeneChip HT HG-U219 perfect-match-only (PM) Array Plate. Scanning was processed in the Gene Titan Platform at our institution, a fully automated array system. Scanned images were analysed with the GeneChip Operating Software (GCOS, Affymetrix). Microarrays data quality assessment was conducted using the Expression Console Software (EC, Affymetrix) and Robust Multichip Analysis algorithm^1^ was used to summarize expression values. Batch effect (by plates) was corrected using mixed model ANOVA algorithms (Partek, USA). Finally, filtering of the batch-adjusted matrix was performed, keeping those probe-sets with expression values larger than 6 in at least 95% of the samples (Partek, USA).

*Array validation by real time PCR*

One μg of RNA was retro-transcribed with random hexamers with the Transcriptor First Strand cDNA synthesis Kit. cDNA was diluted ½ with DEPC water and amplified in triplicate with Taqman assays on demand (Hs00179769_m1, Hs04194186_s1, Hs00428293_m1, Hs00605382_gH, Hs00985031_g1, Hs00180067_m1, Hs00907493_m1, Hs00277097_m1, Hs00964634_m1) in a LightCycler 480 (Roche, Manenheim, Germany). We used Actin (Hs99999903_m1) as a housekeeping gene.

**RESULTS**

*Differential gene expression at baseline and response to smoking*

To investigate if baseline differences in gene expression between groups (S vs. COPD) and/or sex (male vs. female) might influence their transcriptional response to smoking, we investigated if the identified COPD *susceptibility* and *resistance* genes were already DE at baseline between: *(1)* COPD and S, stratified by sex; and, *(2)* males and females, stratified by group.

With respect to the first comparison, we identified 135 that were DE genes at baseline in COPD and S females, and 35 in males (Table E2 presents the top 10 DE genes in both genders). None of them correspond to any of the identified COPD *susceptibility* genes, neither in males or females. By contrast, 10 of the 61 (16%) COPD *resistance* genes identified in females (BCL2, POU2AF1, CDA, ABCG1, MGAM, KCNJ15, CST7, ANXA3, MOSC1, CLEC4D) and one (FGFBP2) out of 16 of those identified in males (6%) were DE already at baseline.

With respect to the second comparison, we found that 64 genes were DE between males and females in COPD, and 72 in S (Table E3 presents the top 10 DE genes in both groups). None of the identified COPD *susceptibility* genes in females (0%) and 1 out of 78 (1%) in males (ARRDC3) were already DE at baseline. On the other hand, 5 out of 61 (8%) identified COPD *resistance* genes in females (FOSL2, DHRS9, MMP9, C20orf3, MME) and 1 out of 16 (6%) in males (SIPA1L2) were already DE basally.

Overall, these observations indicate that differential gene expression at baseline is marginally related to the transcriptomic signature in response to smoking in both groups and genders.

**REFERENCES**

1. Irizarry, R. A., B. M. Bolstad, F. Collin, L. M. Cope, B. Hobbs, and T. P. Speed. 2003. Summaries of Affymetrix GeneChip probe level data. *Nucleic Acids Res* 31:e15.
